# Mechanisms of blood homeostasis: lineage tracking and a neutral model of cell populations in rhesus macaques

**DOI:** 10.1186/s12915-015-0191-8

**Published:** 2015-10-20

**Authors:** Sidhartha Goyal, Sanggu Kim, Irvin SY Chen, Tom Chou

**Affiliations:** Department of Physics, University of Toronto, St George Campus, Toronto, Canada; Department of Microbiology, Immunology, and Molecular Genetics, UCLA, Los Angeles, USA; UCLA AIDS Institute and Department of Medicine, UCLA, Los Angeles, USA; Departments of Biomathematics and Mathematics, UCLA, Los Angeles, USA

**Keywords:** Hematopoiesis, Stem cell clones, Lineage tracking, Mathematical modeling

## Abstract

**Background:**

How a potentially diverse population of hematopoietic stem cells (HSCs) differentiates and proliferates to supply more than 10^11^ mature blood cells every day in humans remains a key biological question. We investigated this process by quantitatively analyzing the *clonal* structure of peripheral blood that is generated by a population of transplanted lentivirus-marked HSCs in myeloablated rhesus macaques. Each transplanted HSC generates a clonal lineage of cells in the peripheral blood that is then detected and quantified through deep sequencing of the viral vector integration sites (VIS) common within each lineage. This approach allowed us to observe, over a period of 4-12 years, hundreds of distinct clonal lineages.

**Results:**

While the distinct clone sizes varied by three orders of magnitude, we found that collectively, they form a steady-state clone size-distribution with a distinctive shape. Steady-state solutions of our model show that the predicted clone size-distribution is sensitive to only two combinations of parameters. By fitting the measured clone size-distributions to our mechanistic model, we estimate both the effective HSC differentiation rate and the number of active HSCs.

**Conclusions:**

Our concise mathematical model shows how slow HSC differentiation followed by fast progenitor growth can be responsible for the observed broad clone size-distribution. Although all cells are assumed to be statistically identical, analogous to a neutral theory for the different clone lineages, our mathematical approach captures the intrinsic variability in the times to HSC differentiation after transplantation.

**Electronic supplementary material:**

The online version of this article (doi:10.1186/s12915-015-0191-8) contains supplementary material, which is available to authorized users.

## Background

Around 10^11^ new mature blood cells are generated in a human every day. Each mature blood cell comes from a unique hematopoietic stem cell (HSC). Each HSC, however, has tremendous proliferative potential and contributes a large number and variety of mature blood cells for a significant fraction of an animal’s life. Traditionally, HSCs have been viewed as a homogeneous cell population, with each cell possessing equal and unlimited proliferative potential. In other words, the fate of each HSC (to differentiate or replicate) would be determined by its intrinsic stochastic activation and signals from its microenvironment [1, 2].

However, as first shown in Muller-Sieburg et al. [3], singly transplanted murine HSCs differ significantly in their long-term lineage (cell-type) output and in their proliferation and differentiation rates [4–7]. Similar findings have been found from examining human embryonic stem cells and HSCs in vitro [8, 9]. While cell-level knowledge of HSCs is essential, it does not immediately provide insight into the question of blood homeostasis at the animal level. More concretely, analysis of single-cell transplants does not apply to human bone marrow transplants, which involve millions of CD34-expressing primitive hematopoietic and committed progenitor cells. Polyclonal blood regeneration from such hematopoietic stem and progenitor cell (HSPC) pools is more complex and requires regulation at both the individual cell and system levels to achieve stable [10, 11] or dynamic [12] homeostasis.

**Fig. 1:**
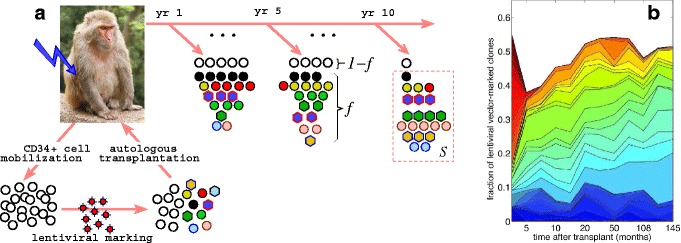
Probing hematopoietic stem and progenitor cell (HSPC) biology through polyclonal analysis. **a** Mobilized CD34+ bone marrow cells from rhesus macaques are first marked individually with lentiviral vectors and transplanted back into the animal after nonlethal myeloablative irradiation [19]. Depending on the animal, 30–160 million CD34+ cells were transplanted, with a fraction ∼0.07–0.3 of them being lentivirus-marked. The clonal contribution of vector-marked HSPCs is measured from blood samples periodically drawn over a dozen years [19]. An average fraction *f* ∼0.03–0.1 of the sampled granulocytes and lymphocytes in the peripheral blood was found to be marked. This fraction is smaller than the fraction of marked CD34+ cells due probably to repopulation by surviving unmarked stem cells in the marrow after myeloablative conditioning. Within any post-transplant sample, *S*=1342–44,415 (average 10,026) viral integration sites of the marked cells were sequenced (see [14, 19] for details). **b** The fraction of all sequenced VIS reads belonging to each clone is shown by the thickness of the slivers. Small clones are not explicitly shown

To dissect how a population of HSPCs supplies blood, several high-throughput assay systems that can quantitatively track repopulation from an individual stem cell have been developed [6, 11, 13, 14]. In the experiment analyzed in this study, as outlined in Fig. 1, each individual CD34+ HSPC is distinctly labeled by the random incorporation of a lentiviral vector in the host genome before transplantation into an animal. All cells that result from proliferation and differentiation of a distinctly marked HSPC will carry identical markings defined by the location of the original viral vector integration site (VIS). By sampling nucleated blood cells and enumerating their unique VISs, one can quantify the cells that arise from a single HSPC marked with a viral vector. Such studies in humans [15] have revealed highly complex polyclonal repopulation that is supported by tens of thousands of different clones [15–18]; a clone is defined as a population of cells of the same lineage, identified here by a unique VIS. These lineages, or clones, can be distributed across all cell types that may be progeny of the originally transplanted HSC after it undergoes proliferation and differentiation. However, the number of cells of any VIS lineage across certain cell types may be different. By comparing abundances of lineages across blood cells of different types, for example, one may be able to determine the heterogeneity or bias of the HSC population or if HSCs often switch their output. This type of analysis remains particularly difficult in human studies since transplants are performed under diseased settings and followed for only 1 or 2 years.

We analyze here a systematic clone-tracking study that used a large number of HSPC clones in a transplant and competitive repopulation setting comparable to that used in humans [19]. In these nonhuman primate rhesus macaque experiments, lentiviral vector-marked clones were followed for up to a decade post-transplantation (equivalent to about 30 years in humans if extrapolated by average life span). All data are available in the supplementary information files of Kim et al. [19]. This long-term study allows one to distinguish clearly HSC clones from other short-term progenitor clones that were included in the initial pool of transplanted CD34+ cells. Hundreds to thousands of detected clones participated in repopulating the blood in a complex yet highly structured fashion. Preliminary examination of some of the clone populations suggests waves of repopulation with short-lived clones that first grow then vanish within the first 1 or 2 years, depending on the animal [19].

Subsequent waves of HSC clones appear to rise and fall sequentially over the next 4–12 years. This picture is consistent with recent observations in a transplant-free transposon-based tagging study in mice [20] and in human gene therapy [15, 16]. Therefore, the dynamics of a clonally tracked nonhuman primate HSPC repopulation provides rich data that can inform our understanding of regulation, stability, HSPC heterogeneity, and possibly HSPC aging in hematopoiesis.

**Fig. 2:**
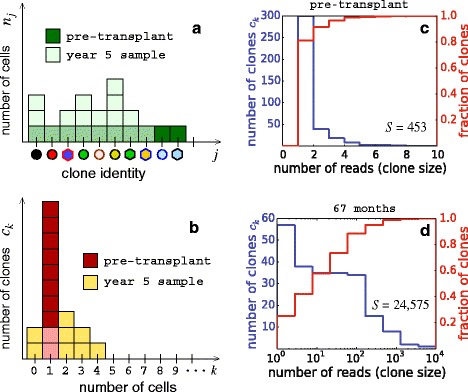
Quantification of marked clones. **a** Assuming each transplanted stem cell is uniquely marked, the initial number of CD34+ cells representing each clone is one. **b** The pre-transplant clone size distribution is thus defined by the total number of transplanted CD34+ cells and is peaked at one cell. Post-transplant proliferation and differentiation of the HSC clones result in a significantly broader clone size distribution in the peripheral blood. The number of differentiated cells for each clone and the number of clones represented by exactly *k* cells, 5 years’ post-transplantation (corresponding to Fig. 1a), are overlaid in (**a**) and (**b**) respectively. **c** Clone size distribution (*blue*) and the cumulative normalized clone size distribution (*red*) of the pre-transplant CD34+ population. **d** After transplantation, clone size distributions in the transit-amplifying (TA) and differentiated peripheral cell pools broaden significantly (with clones ranging over four decades in size) but reach a steady state. The corresponding cumulative normalized distribution is less steep

**Fig. 3:**
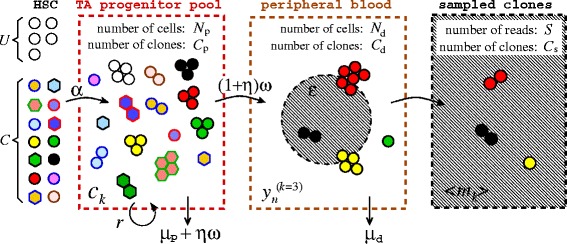
Schematic of our mathematical model. Of the ∼10^6^– 10^7^ CD34+ cells in the animal immediately after transplantation, *C* active HSCs are distinctly labeled through lentiviral vector integration. *U* HSCs are unlabeled because they were not mobilized, escaped lentiviral marking, or survived ablation. All HSCs asymmetrically divide to produce progenitor cells, which in turn replicate with an effective carrying capacity-limited rate *r*. Transit-amplifying progenitor cells die with rate *μ*_p_ or terminally differentiate with rate *ω*. The terminal differentiation of the progenitor cells occurs symmetrically with probability *η* or asymmetrically with probability 1−*η*. This results in a combined progenitor-cell removal rate *μ*=*μ*_p_+*η* *ω*. The differentiated cells outside the bone marrow are assumed not to be subject to direct regulation but undergo turnover with a rate *μ*_d_. The mean total numbers of cells in the progenitor and differentiated populations are denoted *N*_p_ and *N*_d_, respectively. Finally, a small fraction *ε*≪1 of differentiated cells is sampled, sequenced, and found to be marked. In this example, *S*=*ε* *N*_d_=5. Because some clones may be lost as cells successively progress from one pool to the next, the total number of clones in each pool must obey *C*≥*C*_p_≥*C*_d_≥*C*_s_. Analytic expressions for the expected total number of clones in each subsequent pool are derived in Additional file 1. *HSC* hematopoietic stem cell, *TA* transit-amplifying

**Fig. 4:**
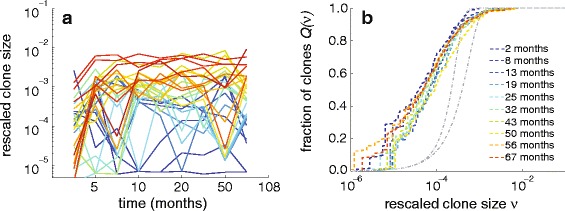
Rescaled and renormalized data. **a** Individual clone populations (here, peripheral blood mononuclear cells of animal RQ5427) show significant fluctuations in time. For clarity, only clones that reach an appreciable frequency are plotted. **b** The corresponding normalized clone size distributions at each time point are rescaled by the sampled and marked fraction of blood, *ν*=*q*/*S*×*f*, where *q* is the number of reads of a particular clone within the sample. After an initial transient, the fraction of clones (*dashed curves*) as a function of relative size remains stable over many years. For comparison, the *dot-dashed gray curves* represent binomial distributions (with *S*=10^3^ and 10^4^ and equivalent mean clone sizes) and underestimate low population clones

Although the time-dependent data from clonal repopulation studies are rich in structure, in this study we focus on one specific aspect of the data: the number of clones that are of a certain abundance as described in Fig. 2. Rather than modeling the highly dynamic populations of each clone, our aim here is to develop first a more global understanding of how the total number of clones represented by specific numbers of cells arises within a mechanistically reasonable model of hematopoiesis. The size distributions of clones present in the blood sampled from different animals at different times are characterized by specific shapes, with the largest clones being a factor of 100–1000 times more abundant than the most rarely detected clones. Significantly, our analysis of renormalized data indicates that the clone size distribution (measuring the number of distinct lineages that are of a certain size) reaches a stationary state as soon as a few months after transplantation (see Fig. 4 below). To reconcile the observed stationarity of the clone size distributions with the large diversity of clonal contributions in the context of HSPC-mediated blood repopulation, we developed a mathematical model that treats three distinct cell populations: HSCs, transit-amplifying progenitor cells, and fully differentiated nucleated blood cells (Fig. 3). While multistage models for a detailed description of differentiation have been developed [21], we lump different stages of cell types within the transit-amplifying progenitor pool into one population, avoiding excess numbers of unmeasurable parameters. Another important feature of our model is the overall effect of feedback and regulation, which we incorporate via a population-dependent cell proliferation rate for progenitor cells.

The effective proliferation rate will be modeled using a Hill-type suppression that is defined by the limited space for progenitor cells in the bone marrow. Such a regulation term has been used in models of cyclic neutropenia [22] but has not been explicitly treated in models of clone propagation in hematopoiesis. Our mathematical model is described in greater detail in the next section and in Additional file 1.

Our model shows that both the large variability and the characteristic shape of the clone size distribution can result from a slow HSC-to-progenitor differentiation followed by a burst of progenitor growth, both of which are generic features of hematopoietic systems across different organisms. By assuming a homogeneous HSC population and fitting solutions of our model to available data, we show that randomness from stochastic activation and proliferation and a global carrying capacity are sufficient to describe the observed clonal structure. We estimate that only a few thousand HSCs may be actively contributing toward blood regeneration at any time. Our model can be readily generalized to include the role of heterogeneity and aging in the transplanted HSCs and provides a framework for quantitatively studying physiological perturbations and genetic modifications of the hematopoietic system.

## Mathematical Model

Our mathematical model explicitly describes three subpopulations of cells: HSCs, transit-amplifying progenitor cells, and terminally differentiated blood cells (see Fig. 3). We will not distinguish between myeloid or lymphoid lineages but will use our model to analyze clone size distribution data for granulocytes and peripheral blood mononuclear cells independently. Our goal will be to describe how clonal lineages, started from distinguishable HSCs, propagate through the amplification and terminal differentiation processes.

Often clone populations are modeled directly by dynamical equations for *n*_*j*_(*t*), the number of cells of a particular clone *j* identified by its specific VIS [23]. Since all cells are identical except for their lentiviral marking, mean-field rate equations for *n*_*j*_(*t*) are identical for all *j*. Assuming identical initial conditions (one copy of each clone), the expected populations *n*_*j*_(*t*) would be identical across all clones *j*. This is a consequence of using identical growth and differentiation *rates* to describe the evolution of the *mean* number of cells of each clone.

Therefore, for cells in any specific pool, rather than deriving equations for the mean number *n*_*j*_ of *cells* of each distinct clone *j* (Fig. 2a), we perform a hodograph transformation [24] and formulate the problem in terms of the number of *clones* that are represented by *k* cells, $c_{k} = \sum _{j}\delta _{k,n_{j}}$ (see Fig. 2b), where the Kronecker *δ* function $\delta _{k,n_{j}}=1$ only when *k*=*n*_*j*_ and is 0 otherwise. This counting scheme is commonly used in the study of cluster dynamics in nucleation [25] and in other related models describing the dynamics of distributions of cell populations. By tracking the number of clones of different sizes, the intrinsic stochasticity in the *times* of cell division (especially that of the first differentiation event) and the subsequent variability in the clone abundances are quantified. Figure 2a, b qualitatively illustrates *n*_*j*_ and *c*_*k*_, pre-transplant and after 5 years, corresponding to the scenario depicted in Fig. 1a. Cells in each of the three pools are depicted in Fig. 3, with different clones grouped according to the number of cells representing each clone.

The first pool (the progenitor-cell pool) is fed by HSCs through differentiation. Regulation of HSC differentiation fate is known to be important for efficient repopulation [26, 27] and control [28] and the balance between asymmetric and symmetric differentiation of HSCs has been studied at the microscopic and stochastic levels [29–32]. However, since HSCs have life spans comparable to that of an animal, we reasoned that the total number of HSCs changes only very slowly after the initial few-month transient after transplant. For simplicity, we will assume, consistent with estimates from measurements [33], that HSCs divide only asymmetrically. Therefore, upon differentiation, each HSC produces one partially differentiated progenitor cell and one replacement HSC. How symmetric HSC division might affect the resulting clone sizes is discussed in Additional file 1 through a specific model of HSC renewal in a finite-sized HSC niche. We find that the incorporation of symmetric division has only a small quantitative effect on the clone size distribution that we measure and ultimately analyze.

Next, consider the progenitor-cell pool. From Fig. 3, we can count the number of clones *c*_*k*_ represented by exactly *k* cells. For example, the black, red, green, and yellow clones are each represented by three cells, so *c*_3_=4. Each progenitor cell can further differentiate with rate *ω* into a terminally differentiated cell. If progenitor cells undergo symmetric differentiation with probability *η* and asymmetric differentiation with probability 1−*η*, the effective rate of differentiation is 2*ηω*+(1−*η*)*ω*=(1+*η*)*ω*. In turn, fully differentiated blood cells (not all shown in Fig. 3) are cleared from the peripheral pool at rate *μ*_d_, providing a turnover mechanism. Finally, each measurement is a small-volume sample drawn from the peripheral blood pool, as shown in the final panel in Fig. 3.

Note that the transplanted CD34+ cells contain both true HSCs and progenitor cells. However, we assume that at long times, specific clones derived from progenitor cells die out and that only HSCs contribute to long-lived clones. Since we measure the number of clones of a certain size rather than the dynamics of individual clone numbers, transplanted progenitor cells should not dramatically affect the steady-state clone size distribution. Therefore, we will ignore transplanted progenitor cells and assume that after transplantation, effectively only *U* unlabeled HSCs and *C* labeled (lentivirus-marked) HSCs are present in the bone marrow and actively asymmetrically differentiating (Fig. 3). Mass-action equations for the expected number of clones *c*_*k*_ of size *k* are derived from considering simple birth and death processes with immigration (HSC differentiation):

(1) $$ \begin{aligned} \frac{\mathrm{d} c_{k}}{\mathrm{d} t} = \underbrace{ \alpha\left[c_{k-1} - c_{k}\right]}_{\textrm{HSC differentiation}} &+ \underbrace{r\left[(k-1)c_{k-1}-{kc}_{k}\right]}_{\textrm{progenitor birth}}\\ &+ \underbrace{\mu\left[(k+1)c_{k+1} - k c_{k}\right]}_{\textrm{progenitor death}},  \end{aligned}  $$

where *k*=1,2,…,*C* and $c_{0}(t) \equiv C - \sum _{k=1}^{\infty }c_{k}(t)$ is the number of clones that are not represented in the progenitor pool. Since *C* is large, and the number of clones that are of size comparable to *C* is negligible, we will approximate *C*→*∞* in our mathematical derivations. We have suppressed the time dependence of *c*_*k*_(*t*) for notational simplicity. The constant parameter *α* is the asymmetric differentiation rate of all HSCs, while *r* and *μ* are the proliferation and overall clearance rates of progenitor cells. In our model, HSC differentiation events that feed the progenitor pool are implicitly a rate- *α* Poisson process. The appreciable number of detectable clones (Fig. 1b) implies the initial number *C* of HSC clones is large enough that asymmetric differentiation of individual HSCs is uncorrelated. The alternative scenario of a few HSCs undergoing synchronized differentiation would not lead to appreciably different results since the resulting distribution *c*_*k*_ is more sensitive to the progenitor cells’ *unsynchronized* replication and death than to the statistics of the immigration by HSC differentiation.

The final differentiation from progenitor cell to peripheral blood cell can occur through symmetric or asymmetric differentiation, with probabilities *η* and 1−*η*, respectively. If parent progenitor cells are unaffected after asymmetric terminal differentiation (i.e., they die at the normal rate *μ*_p_), the dynamics are feed-forward and the progenitor population is not influenced by terminal differentiation. Under symmetric differentiation, a net loss of one progenitor cell occurs. Thus, the overall progenitor-cell clearance rate can be decomposed as *μ*=*μ*_p_+*ηω*. We retain the factor *η* in our equations for modeling pedagogy, although in the end it is subsumed in effective parameters and cannot be independently estimated from our data.

The first term in Eq. 1 corresponds to asymmetric differentiation of each of the *C* active clones, of which *c*_*k*_ are of those lineages with population *k* already represented in the progenitor pool. Differentiation of this subset of clones will add another cell to these specific lineages, reducing *c*_*k*_. Similarly, differentiation of HSCs in lineages that are represented by *k*−1 progenitor cells adds cells to these lineages and increases *c*_*k*_. Note that Eq. 1 are mean-field rate equations describing the evolution of the expected number of clones of size *k*. Nonetheless, they capture the intrinsic dispersion in lineage sizes that make up the clone size distribution. While all cells are assumed to be statistically identical, with equal rates *α*, *p*, and *μ*, Eq. 1 directly model the evolution of the *distributionc*_*k*_(*t*) that arises ultimately from the distribution of times for each HSC to differentiate or for the progenitor cells to replicate or die. Similar equations have been used to model the evolving distribution of virus capsid sizes [34].

Since the equations for *c*_*k*_(*t*) describe the evolution of a distribution, they are sometimes described as master equations for the underlying process [34, 35]. Here we note that the solution to Eq. 1, *c*_*k*_(*t*), is the *expected* distribution of clone sizes. Another level of stochasticity could be used to describe the evolution of a *probability distribution*$P_{b}(\textbf {b};t) = P_{b}(b_{0}, b_{1},\ldots,b_{N_{\mathrm {p}}};t)\phantom {\dot {i}\!}$*over the integer numbersb*_*k*_. This density represents the *probability* that at time *t*, there are *b*_0_ unrepresented lineages, *b*_1_ lineages represented by one cell in the progenitor pool, *b*_2_ lineages represented by two cells in the progenitor pool, and so on. Such a probability distribution would obey an *N*_p_-dimensional master equation rather than a one-dimensional equation, like Eq. 1, and once known, can be used to compute the mean $c_{k}(t) = \sum _{\textbf {b}} b_{k}P(\textbf {b};t)$. To consider the entire problem stochastically, the variability described by probability distribution *P*_*b*_ would have to be propagated forward to the differentiated cell pool as well. Given the modest number of measured data sets and the large numbers of lineages that are detectable in each, we did not attempt to use the data as samples of the distribution *P*_*b*_ and directly model the mean values *c*_*k*_ instead. Variability from both intrinsic stochasticity and sampling will be discussed in Additional file 1.

After defining *u*(*t*) as the number of unlabeled cells in the progenitor pool, and $N_{\mathrm {p}}(t) = u(t)+\sum _{k=1}^{\infty }{kc}_{k}(t)$ as the total number of progenitor cells, we find $\dot {u} = (r - \mu) u + \alpha U$ and

(2) $$ \frac{\mathrm{d} N_{\mathrm{p}}(t)}{\mathrm{d} t} = \alpha \left(U+C\right)+\left(r-\mu \right)N_{\mathrm{p}}(t).   $$

Without regulation, the total population *N*_p_(*t*→*∞*) will either reach *N*_p_≈*α*(*U*+*C*)/(*μ*−*r*) for *μ*>*r* or will exponentially grow without bound for *r*>*μ*. Complex regulation terms have been employed in deterministic models of differentiation [28] and in stochastic models of myeloid/lymphoid population balance [36]. For the purpose of estimating macroscopic clone sizes, we assume regulation of cell replication and/or spatial constraints in the bone marrow can be modeled by a simple effective Hill-type growth law [22, 37]:

(3) $$ r = r(N_{\mathrm{p}}) \equiv \frac{pK}{N_{\mathrm{p}}+K}   $$

where *p* is the intrinsic replication rate of an isolated progenitor cell. We assume that progenitor cells at low density have an overall positive growth rate *p*>*μ*. The parameter *K* is the progenitor-cell population in the bone marrow that corresponds to the half-maximum of the effective growth rate. It can also be interpreted as a limit to the bone marrow size that regulates progenitor-cell proliferation to a value determined by *K*, *p*, and *μ* and is analogous to the carrying capacity in logistic models of growth [38]. For simplicity, we will denote *K* as the carrying capacity in Eq. 3 as well. Although our data analysis is insensitive to the precise form of regulation used, we chose the Hill-type growth suppression because it avoids negative growth rates that confuse physiological interpretation. An order-of-magnitude estimate of the bone marrow size (or carrying capacity) in the rhesus macaque is *K*∼10^9^. Ultimately, we are interested in how a limited progenitor pool influences the overall clone size distribution, and a simple, single-parameter (*K*) approximation to the progenitor-cell growth constraint is sufficient.

Upon substituting the growth law *r*(*N*_p_) described by Eq. 3 into Eq. 2, the total progenitor-cell population *N*_p_(*t*→*∞*) at long times is explicitly shown in Additional file 1: Eq. A19 to approach a finite value that depends strongly on *K*. Progenitor cells then differentiate to supply peripheral blood at rate (1+*η*)*ω* so that the total number of differentiated blood cells obeys

(4) $$ \frac{\mathrm{d} N_{\mathrm{d}}(t)}{\mathrm{d} t} = (1+\eta)\omega N_{\mathrm{p}} - \mu_{\mathrm{d}}N_{\mathrm{d}}.   $$

At steady state, the combined peripheral nucleated blood population is estimated to be *N*_d_∼10^9^– 10^10^ [39], setting an estimate of *N*_d_/*N*_p_≈(1+*η*)*ω*/*μ*_d_∼1–10. Moreover, as we shall see, the relevant factor in our steady-state analysis will be the numerical *value* of the effective growth rate *r*, rather than its functional form. Therefore, the chosen form for regulation will not play a role in the mathematical results in this paper except to define parameters (such as *K*) explicitly in the regulation function itself.

To distinguish and quantify the clonal structure within the peripheral blood pool, we define $y_{n}^{(k)}$ to be the number of clones that are represented by exactly *n* cells in the differentiated pool *andk* cells in the progenitor pool. For example, in the peripheral blood pool shown in Fig. 3, $y_{1}^{(3)} = y_{2}^{(3)} = y_{4}^{(3)} = y_{6}^{(3)} = 1$. This counting of clones across both the progenitor and peripheral blood pools is necessary to balance progenitor-cell differentiation rates with peripheral blood turnover rates. The evolution equations for $y_{n}^{(k)}$ can be expressed as

(5) $$ \frac{\mathrm{d} y_{n}^{(k)}}{\mathrm{d} t} = (1+\eta)\omega k \left(y_{n-1}^{(k)} - y_{n}^{(k)}\right) + (n+1) \mu_{\mathrm{d}}y_{n+1}^{(k)} - n \mu_{d} y_{n}^{(k)},   $$

where $y_{0}^{(k)} \equiv c_{k} - \sum _{n=1}^{\infty }y_{n}^{(k)}$ represents the number of progenitor clones of size *k* that have not yet contributed to peripheral blood. The transfer of clones from the progenitor population to the differentiated pool arises through $y_{0}^{(k)}$ and is simply a statement that the number of clones in the peripheral blood can increase only by differentiation of a progenitor cell whose lineage has not yet populated the peripheral pool. The first two terms on the right-hand side of Eq. 5 represent immigration of clones represented by *n*−1 and *n* differentiated cells *conditioned upon* immigration from only those specific clones represented by *k* cells in the progenitor pool. The overall rate of addition of clones from the progenitor pool is thus (1+*η*)*ωk*, in which the frequency of terminal differentiation is weighted by the stochastic division factor (1+*η*). By using the Hill-type growth term *r*(*N*_p_) from Eq. 3, Eq. 1 can be solved to find *c*_*k*_(*t*), which in turn can be used in Eq. 5 to find $y_{n}^{(k)}(t)$. The number of clones in the peripheral blood represented by exactly *n* differentiated cells is thus $y_{n}(t) = \sum _{k=1}^{\infty }y_{n}^{(k)}(t)$.

As we mentioned, Eqs. 1 and 5 describe the evolution of the expected clone size distribution. Since each measurement represents one realization of the distributions *c*_*k*_(*t*) and *y*_*n*_(*t*), the validity of Eqs. 1 and 5 relies on a sufficiently large *C* such that the marked HSCs generate enough lineages and cells to allow the subsequent peripheral blood clone size distribution to be sampled adequately. In other words, measurement-to-measurement variability described by e.g., $\phantom {\dot {i}\!}\langle c_{k}(t)c_{k^{\prime }}(t)\rangle - \langle c_{k}(t)\rangle \langle c_{k^{\prime }}(t)\rangle $ is assumed negligible (see Additional file 1). Our modeling approach would not be applicable to studying single HSC transplant studies [4–6] unless the measured clone sizes from multiple experiments are aggregated into a distribution.

Finally, to compare model results with animal blood data, we must consider the final step of sampling small aliquots of the differentiated blood. As derived in Additional file 1: Eq. A11, if *S* marked cells are drawn and sequenced successfully (from a total differentiated cell population *N*_d_), the expected number of clones 〈*m*_*k*_(*t*)〉 represented by *k* cells is given by

(6) $$ \begin{array}{cc}\left\langle {m}_k(t)\right\rangle & =F\left(q,t\right)-F\left(q-1,t\right)\\ {}=\sum_{\ell =0}^{\infty }{\mathrm{e}}^{-\ell \varepsilon}\frac{{\left(\ell \varepsilon \right)}^k}{k!}{y}_{\ell }(t),\end{array} $$

where *ε*≡*S*/*N*_d_≪1 and $F(q,t) \equiv \sum _{k=0}^{q}\langle m_{k}(t)\rangle $ is the sampled, expected cumulative size distribution. Upon further normalization by the total number of detected clones in the sample, *C*_s_(*t*)=*F*(*S*,*t*)−*F*(0,*t*), we define

(7) $$ Q(q,t) \equiv \frac{F(q, t) - F(0,t)}{F(S,t)-F(0,t)}   $$

as the fraction of the total number of sampled clones that are represented by *q* or fewer cells. Since the data represented in terms of *Q* will be seen to be time-independent, explicit expressions for $c_{k}, y_{n}^{(k)}$, 〈*m*_*k*_〉, and *Q*(*q*) can be derived. Summarizing, the main features and assumptions used in our modeling include:

- A neutral-model framework [40] that directly describes the distribution of clone sizes in each of the three cell pools: progenitor cells, peripheral blood cells, and sampled blood cells. The cells in each pool are statistically identical.
- A constant asymmetric HSC differentiation rate *α*. The appreciable numbers of unsynchronized HSCs allow the assumption of Poisson-distributed differentiation times of the HSC population. The level of differentiation symmetry is found to have little effect on the steady-state clone size distribution (see Additional file 1). The symmetry of the terminal differentiation step is also irrelevant for understanding the available data.
- A simple one-parameter (*K*) growth regulation model that qualitatively describes the finite maximum size of the progenitor population in the bone marrow. Ultimately, the specific form for the regulation is unimportant since only the steady-state value of the growth parameter *r* affects the parameter fitting.

Using only these reasonable model features, we are able to compute clone size distributions and compare them with data. An explicit form for the expected steady-state clone size distribution 〈*m*_*k*_〉 is given in Additional file 1: Eq. A32, and the parameters and variables used in our analysis are listed in Table 1.

**Table 1:** Model parameters and variables. Estimates of steady-state values are provided where available. We assume little prior knowledge on all but a few of the more established parameters. Nonetheless, our modeling and analysis place constraints on combinations of parameters, allowing us to fit data and provide estimates for steady-state values of *U*+*C*∼10^3^– 10^4^ and *α*(*N*_p_+*K*)/(*p* *K*)∼0.002–0.1

Symbol	Parameter or variable	Estimate	Ref.
*α*	Single HSC asymmetric differentiation rate	∼0.1–0.3 per month	[46, 51]
*p*	Free progenitor-cell proliferation rate		
*μ*_p_	Progenitor-cell death rate		
*μ*_d_	Differentiated cell death rate	∼0.01–0.3 per day	[52, 53]
*η*	Symmetric differentiation probability		
*ω*	Terminal differentiation rate		
*K*	Progenitor-cell capacity	∼10^9^	
*U*	Number of active unmarked HSCs		
*C*	Number of active viral-marked HSCs		
*C*_p_	Number of progenitor clones		Additional file 1: Eq. A23
*C*_d_	Number of differentiated clones		Additional file 1: Eq. A23
*C*_s_	Number of sampled clones		Additional file 1: Eq. A24
*S*	Number of sequences read	∼10^3^– 10^4^	[14]
*c*_*k*_	Number of progenitor clones of size *k*		
*u*	Unlabeled progenitor-cell population		
*N*_p_	Total progenitor-cell population		
*N*_d_	Total differentiated blood population	∼10^9^– 10^10^	
$y_{n}^{(k)}$	Number of differentiated clones of size *n* arising from progenitors of size *k*		

## Results and discussion

In this section, we describe how previously published data (the number of cells of each detected clone in a sample of the peripheral blood, which are available in the supplementary information files of Kim et al. [19]) are used to constrain parameter values in our model. We emphasize that our model is structurally different from models used to track lineages and clone size distributions in retinal and epithelial tissues [41, 42]. Rather than tracking only the lineages of stem cells (which are allowed to undergo asymmetric differentiation, symmetric differentiation, or symmetric replication), our model assumes a highly proliferative population constrained by a carrying capacity *K* and slowly fed at rate *α* by an asymmetrically dividing HSC pool of *C* fixed clones. We have also included terminal differentiation into peripheral blood and the effects of sampling on the expected clone size distribution. These ingredients yield a clone size distribution different from those previously derived [41, 42], as described in more detail below.

### Stationarity in time

Clonal contributions of the initially transplanted HSC population have been measured over 4–12 years in four different animals. As depicted in Fig. 4a, populations of individual clones of peripheral blood mononuclear cells from animal RQ5427, as well as all other animals, show significant variation in their dynamics. Since cells of any detectable lineage will number in the millions, this variability in lineage size across time cannot be accounted for by the intrinsic stochasticity of progenitor-cell birth and death. Rather, these rises and falls of lineages likely arise from a complicated regulation of HSC differentiation and lineage aging. However, in our model and analysis, we do not keep track of lineage sizes *n*_*i*_. Instead, define *Q*(*ν*) as the fraction of clones arising with relative frequency *ν*≡*fq*/*S* or less (here, *q* is the number of VIS reads of any particular clone in the sample, *f* is the fraction of all sampled cells that are marked, and *S* is the total number of sequencing reads of marked cells in a sample). Figure 4b shows data analyzed in this way and reveals that *Q*(*ν*) appears stationary in time.

The observed steady-state clone size distribution is broad, consistent with the mathematical model developed above. The handful of most populated clones constitutes up to 1–5 % of the entire differentiated blood population. These dominant clones are followed by a large number of clones with fewer cells. The smallest clones sampled in our experiment correspond to a single read *q*=1, which yields a minimum measured frequency *ν*_min_=*f*/*S*. A single read may comprise only 10^−4^– 10^−3^ % of all differentiated blood cells. Note that the cumulative distribution *Q*(*ν*) exhibits higher variability at small sizes simply because fewer clones lie below these smaller sizes.

Although engraftment occurs within a few weeks and total blood populations *N*_p_ and *N*_d_ (and often immune function) re-establish themselves within a few months after successful HSC transplant [43, 44], it is still surprising that the clone size distribution is relatively static within each animal (see Additional file 1 for other animals). Given the observed stationarity, we will use the steady-state results of our mathematical model (explicitly derived in Additional file 1) for fitting data from each animal.

### Implications and model predictions

By using the exact steady-state solution for *c*_*k*_ (Additional file 1: Eq. A21) in Additional file 1: Eq. A18, we can explicitly evaluate the expected clone size distribution 〈*m*_*k*_〉 using Eq. 6, and the expected cumulative clone fraction *Q*(*q*) using Eq. 7. In the steady state, the clone size distribution of progenitor cells can also be approximated by a gamma distribution with parameters *a*≡*α*/*r* and $\bar {r} \equiv r/\mu $: $c_{k} \sim \bar {r}^{k} k^{-1+a}$ (see Additional file 1: Eq. A27). In realistic steady-state scenarios near carrying capacity, *r*=*r*(*N*_p_)≲*μ*, as calculated explicitly in Additional file 1: Eq. A20. By defining $\bar {r}=r/\mu = 1-\delta $, we find that *δ* is inversely proportional to the carrying capacity:

(8) $$ \delta \approx \frac{\alpha}{\mu} \frac{\mu}{p-\mu} \frac{U+C}{K} \ll 1.   $$

**Fig. 5:**
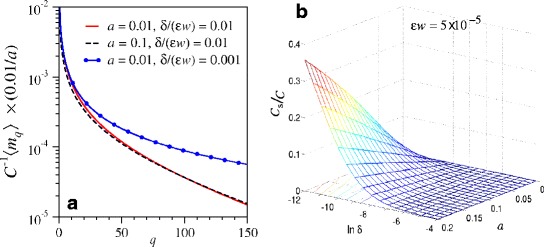
Clone size distributions and total number of sampled clones. **a** Expected clone size distributions *C* ^−1^〈*m*_*q*_〉 derived from the approximation in Additional file 1: Eq. A32 are plotted for various *a* and *δ*/(*ε* *w*) [where *w*≡(1+*η*)*ω*/*μ*_d_]. The nearly coincident *solid* and *dashed curves* indicate that variations in *a* mainly scale the distribution by a multiplicative factor. In contrast, the combination *δ*/(*ε* *w*) controls the weighting at large clone sizes through the population cut-off imposed by the carrying capacity. Of the two controlling parameters, the steady-state clone size distribution is most sensitive to *R*≅*δ*/(*ε* *w*). The dependence of data-fitting on these two parameters is derived in Additional file 1 and discussed in the next section. **b** For *ε* *w*=5×10^−5^, the expected fraction *C*_s_/*C* of active clones sampled as a function of ln*δ* and *α*. The expected number of clones sampled increases with carrying capacity *K*, HSC differentiation rate *a*=*α*/*r*, and the combined sampling and terminal differentiation rate *ε* *w*

The dependencies of 〈*m*_*q*_〉 on *δ* and *a*=*α*/*r* are displayed in Fig. 5a, in which we have defined *w*≡(1+*η*)*ω*/*μ*_d_.

Although our equations form a mean-field model for the expected number of measured clones of any given size, randomness resulting from the stochastic differentiation times of individual HSCs (all with the same rate *α*) is taken into account.

This is shown in Additional file 1: Eqs. A36–A39, where we explicitly consider the fully stochastic population of a single progenitor clone that results from the differentiation of a single HSC. Since independent unsynchronized HSCs differentiate at times that are exponentially distributed (with rate *α*), we construct the expected clone size distribution from the birth–death–immigration process [45] to find a result equivalent to that derived from our original model (Eq. 1 and Additional file 1: Eq. A21). Thus, we conclude that if *a*=*α*/*r* is small, the shape of the expected clone size distribution is mainly determined at short times by the initial repopulation of the progenitor-cell pool.

Our model also suggests that the expected number of sampled clones relative to the number of active transplanted clones (see Additional file 1: Eq. A24) can be expressed as:

(9) $$ \begin{aligned} \frac{C_{\mathrm{s}}}{C} & \approx \left[1-\left(\frac{\delta}{1-(1-\delta)e^{-\varepsilon w}}\right)^{a}\right] \\ & \approx \frac{\alpha}{r}\ln \left(\frac{\varepsilon w}{\delta}+1\right),  \end{aligned}  $$

where the last approximation is accurate for *εw*≪1 and *C*_s_/*C*≪1. The clonal diversity one expects to measure in the peripheral blood sample is sensitive to the combination of biologically relevant parameters and rates *δ* and *a*=*α*/*r*. Figure 5b shows the explicit dependence of the fraction of active clones on *a* and the combination of parameters defining *δ*, for *εw*=*ε*(1+*η*)*ω*/*μ*_d_=5×10^−5^.

Our analysis shows how scaled measurable quantities such as *C*_s_/*C* and *C*^−1^〈*m*_*q*_〉 depend on just a few combinations of experimental and biological parameters. This small domain of parameter sensitivity reduces the number of parameters that can be independently extracted from clone size distribution data. For example, the mode of terminal differentiation described by *η* clearly cannot be extracted from clonal tracking measurements. Similarly, models that are more detailed of the complex regulation processes would introduce additional parameters that are not resolved by these experiments. Nonetheless, we shall fit our data and known information contained in the experimental protocol to our model to estimate biologically relevant parameters, such as the total number of activated HSCs *U*+*C*, and thus indirectly *C*.

### Model fitting

Our mathematical model for 〈*m*_*k*_〉 (and *F*(*q*) and *Q*(*q*)) includes numerous parameters associated with the processes of HSC differentiation, progenitor-cell amplification, progenitor-cell differentiation, peripheral blood turnover, and sampling. Data fitting is performed using clone size distributions derived separately from the read counts from both the left and right ends of each VIS (see [14] for details on sequencing). Even though we fit our data to 〈*m*_*k*_〉 using three independent parameters, *a*=*α*/*r*, $\bar {r}= r/\mu $, and *εw*, we found that within the relevant physiological regime, all clone distributions calculated from our model are most sensitive to just two combinations of parameters (see Additional file 1 for an explicit derivation):

(10) $$ a \equiv \frac{\alpha}{r}\quad \text{and} \quad R \equiv \frac{\varepsilon w}{\ln \left(1/\bar{r}\right)}\approx \frac{\varepsilon w}{\delta} = \frac{(1+\eta)\omega S}{N_{\mathrm{d}}\mu_{\mathrm{d}}\delta},   $$

where the last approximation for *R* is valid when $1-\bar {r} = \delta \ll 1$. While the fits are rather insensitive to *εw* this parameter can fortunately be approximated from estimates of *S* and the typical turnover rate of differentiated blood. Consequently, we find two maximum likelihood estimates (MLEs) for *a* and *R* at each time point. It is important to note that fitting our model to steady-state clone size distributions does not determine all of the physiological parameters arising in our equations. Rather, they provide only two constraints that allow one to relate their values.

**Fig. 6:**
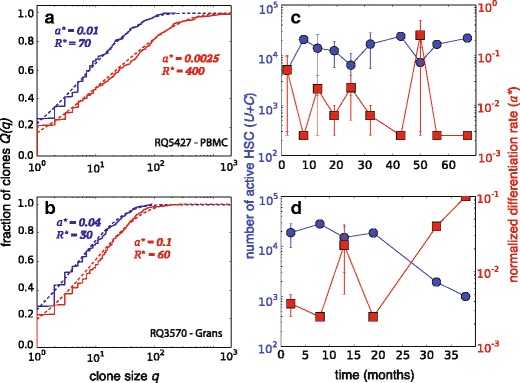
Data fitting. **a** Fitting raw (not rescaled, as shown in Figure 4) clone size distribution data to 〈*m*_*k*_〉 from Eq. 6 at two time points for animal RQ5427. The maximum likelihood estimates (MLEs) are (*a* ^∗^≈0.01,*R* ^∗^≈70) and (*a* ^∗^≈0.0025,*R* ^∗^≈400) for data taken at 32 (*blue*) and 67 (*red*) months post-transplant, respectively. Note that the MLE values for different samples vary primarily due to different values of *S* (and hence *ε*) used in each measurement. **b** For animal RQ3570, the clone fractions at 32 (*blue*) and 38 (*red*) months yield (*a* ^∗^≈0.04,*R* ^∗^≈30) and (*a* ^∗^≈0.1,*R* ^∗^≈60), respectively. For clarity, we show the data and fitted models in terms of *Q*(*q*). **c** Estimated number of HSCs *U*+*C* (*circles*) and normalized differentiation rate *a* (*squares*) for animal RQ5427. **d** *U*+*C* and *a* for animal RQ3570. Note the temporal variability (but also long-term stability) in the estimated number of contributing HSCs. Additional details and fits for other animals are qualitatively similar and given in Additional file 1. *HSC* hematopoietic stem cell, *PBMC*, peripheral blood mononuclear cell Grans, granulocytes

For ease of presentation, henceforth we will show all data and comparisons with our model equations in terms of the fraction *Q*(*ν*) or *Q*(*q*) (Figs. 4b and 6a, b). Figure 6a, b shows MLE fitting to the raw data 〈*m*_*k*_〉 plotted in terms of the normalized but unrescaled data *Q*(*q*) for two different peripheral blood cell types from two animals (RQ5427 and RQ3570). Data from all other animals are shown and fitted in Additional file 1, along with overall goodness-of-fit metrics. Raw cell count data are given in Kim et al. [19].

### HSC asymmetric differentiation rate

The MLE for *a*=*α*/*r*, *a*^∗^, was typically in the range 10^−2^– 10^−1^. Given realistic parameter values, this quantity mostly provides an estimate of the HSC relative differentiation rate *a*^∗^∼*α*/(*μ*_p_+*ηω*). The smallness of *a*^∗^ indicates slow HSC differentiation relative to the progenitor turnover rate *μ*_p_ and the final differentiation rate *ηω*, consistent with the dominant role of progenitor cells in populating the total blood tissue. Note that besides the intrinsic insensitivity to *εw*, the goodness-of-fit is also somewhat insensitive to small values of *a*^∗^ due to the weak dependence of *c*_*k*_∼1/*k*^1−*a*^ on *a* (see Additional file 1). The normalized relative differentiation rates estimated from two animals are shown by the squares (right axis) in Fig. 6c, d.

### Number of HSCs

The stability of blood repopulation kinetics is also reflected in the number of estimated HSCs that contribute to blood (shown in Fig. 6c, d). The total number of HSCs is estimated by expressing *U*+*C* in terms of the effective parameters, *R* and *a*, which in turn are functions of microscopic parameters (*α*,*p*,*μ*_p_,*μ*_d_,*w*, and *K*) that cannot be directly measured. In the limit of small sample size, *S*≪*R*^∗^*K*, however, we find *U*+*C*≈*S*/(*R*^∗^*a*^∗^) (see Additional file 1), which can then be estimated using the MLEs *a*^∗^ and *R*^∗^ obtained by fitting the clone size distributions. The corresponding values of *U*+*C* for two animals are shown by the circles (left axis) in Fig. 6c, d. Although variability in the MLEs exists, the fluctuations appear stationary over the course of the experiment for each animal (see Additional file 1).

## Conclusions

Our clonal tracking analysis revealed that individual clones of HSCs contributed differently to the final differentiated blood pool in rhesus macaques, consistent with mouse and human data. Carefully replotting the raw data (clone sizes) in terms of the normalized, rescaled cumulative clone size distribution (the fraction of all detected clones that are of a certain size or less) shows that these distributions reach steady state a few months after transplantation. Our results carry important implications for stem cell biology. Maintaining homeostasis of the blood is a critical function for an organism. Following a myeloablative stem cell transplant, the hematopoietic system must repopulate rapidly to ensure the survival of the host. Not only do individual clones rise and fall temporally, as previously shown [19], but as any individual clone of a certain frequency declines, it is replaced by another of similar frequency. This exchange-correlated mechanism of clone replacement may provide a mechanism by which overall homeostasis of hematopoiesis is maintained long term, thus ensuring continued health of the blood system.

To understand these observed features and the underlying mechanisms of stem cell-mediated blood regeneration, we developed a simple neutral population model of the hematopoietic system that quantifies the dynamics of three subpopulations: HSCs, transit-amplifying progenitor cells, and fully differentiated nucleated blood cells. We also include the effects of global regulation by assuming a Hill-type growth rate for progenitor cells in the bone marrow but ignore cell-to-cell variation in differentiation and proliferation rates of all cells.

Even though we do not include possible HSC heterogeneity, variation in HSC activation, progenitor-cell regulation, HSC and progenitor-cell aging (progenitor bursting), niche- and signal molecule-mediated controls, or intrinsic genetic and epigenetic differences, solutions to our simple *homogeneous* HSC model are remarkably consistent with observed clone size distributions. As a first step, we focus on how the intrinsic stochasticity in just the cellular birth, death, and differentiation events gives rise to the progenitor clone size distribution.

To a large extent, the exponentially distributed first HSC differentiation times and the growth and turnover of the progenitor pool control the shape of the expected long-time clone size distribution. Upon constraining our model to the physiological regime relevant to the experiments, we find that the calculated shapes of the clone size distributions are sensitive to effectively only two composite parameters. The HSC differentiation rate *α* sets the scale of the expected clone size distribution but has little effect on the shape. Parameters, including carrying capacity *K*, active HSCs *U*+*C*, and birth and death rates *p*,*ω*,*μ*_p_,*μ*_d_, influence the shape of the expected clone size distribution 〈*m*_*q*_〉 only through the combination *R*, and only at large clone sizes.

Our analysis allowed us to estimate other combinations of model parameters quantitatively. Using a MLE, we find values for the effective HSC differentiation rate *a*^∗^∼10^−2^– 10^−1^ and the number of HSCs that are contributing to blood within any given time frame *U*+*C*∼10^3^– 10^4^. Since the portion of HSCs that contribute to blood may vary across their typical life span *L*∼25 years, the total number of HSCs can be estimated by (*U*+*C*)×*L*/*τ*, where *τ*∼1 year [19]. Our estimate of a total count of ∼3×10^4^– 3×10^5^ HSCs is about 30-fold higher than the estimate of Abkowitz et al. [33] but is consistent with Kim et al. [19]. Note that the ratio of *C* to the total number of initially transplanted CD34+ cells provides a measure of the overall potency of the transplant towards blood regeneration. In the extreme case in which one HSC is significantly more potent (through, e.g., a faster differentiation rate), this ratio would be smaller. An example of this type of heterogeneity would be an HSC with one or more cancer-associated mutations, allowing it to out-compete other transplanted normal HSCs. Hence, our clonal studies and the associated mathematical analysis can provide a framework for characterizing normal clonal diversity as well as deviations from it, which may provide a metric for early detection of cancer and other related pathologies.

Several simplifying assumptions have been invoked in our analysis. Crucially, we assumed HSCs divided only asymmetrically and ignored instances of symmetric self-renewal or symmetric differentiation. The effects of symmetric HSC division can be quantified in the steady-state limit. In previous studies, the self-renewal rate for HSCs in primates is estimated as 4–9 months [46, 47], which is slightly longer than the short timescale (∼2–4 months) on which we observe stabilization of the clone size distribution. Therefore, if the HSC population slowly increases in time through occasional symmetric division, the clone size distribution in the peripheral blood will also shift over long times. The static nature of the clone distributions over many years suggests that size distributions are primarily governed by mechanisms operating at shorter timescales in the progenitor pool. For an HSC population (such as cancerous or precancerous stem cells [48]) that has already expanded through early replication, the initial clone size distribution within the HSC pool can be quantified by assuming an HSC pool with separate carrying capacity *K*_HSC_. Such an assumption is consistent with other analyses of HSC renewal [49]. All our results can be used (with the replacement *C*→*K*_HSC_) if the number of transplanted clones *C*≥*K*_HSC_ because replication is suppressed in this limit. When *K*_HSC_≫*C*≫1, replicative expansion generates a broader initial HSC clone size distribution (see Additional file 1). The resulting final peripheral blood clone size distribution can still be approximated by our result (Eq. 6) if the normalized differentiation rate *a*≪1, exhibiting the insensitivity of the differentiated clone size distribution to a broadened clone size distribution at the HSC level. However, if HSC differentiation is sufficiently fast (*a*≪̸1), the clonal distribution in the progenitor and differentiated pools may be modified.

To understand the temporal dynamics of clone size distributions, a more detailed numerical study of our full time-dependent neutral model is required. Such an analysis can be used to investigate the effects of rapid temporal changes in the HSC division mode [41]. Temporal models would also allow investigation into the evolution of HSC mutations and help unify concepts of clonal stability (as indicated by the stationarity of rescaled clone size distributions) with ideas of clonal succession [10, 11] or dynamic repetition [12] (as indicated by the temporal fluctuations in the estimated number *U*+*C* of active HSCs). Predictions of the time-dependent behavior of clone size distributions will also prove useful in guiding future experiments in which the animals are physiologically perturbed via e.g., myeloablation, hypoxiation, and/or bleeding. In such experimental settings, regulation may also occur at the level of HSC differentiation (*α*) and a different mathematical model may be more appropriate.

We have not addressed the temporal fluctuations in *individual* clone abundances evident in our data (Fig. 4a) or in the wave-like behavior suggested by previous studies [19]. Since the numbers of detectable cells of each VIS lineage in the whole animal are large, we believe these fluctuations do not arise from intrinsic cellular stochasticity or sampling. Rather, they likely reflect slow timescale HSC transitions between quiescent and active states and/or HSC aging [50]. Finally, subpopulations of HSCs that have different intrinsic rates of proliferation, differentiation, or clearance could then be explicitly treated. As long as each subtype in a heterogeneous HSC or progenitor-cell population does not convert into another subtype, the overall aggregated clone size distribution 〈*m*_*k*_〉 will preserve its shape. Although steady-state data are insufficient to provide resolution of cell heterogeneity, more resolved temporal data may allow one to resolve different parameters associated with different cell types. Such extensions will allow us to study the temporal dynamics of individual clones and clone populations in the context of cancer stem cells and will be the subject of future work.

## Additional file

Additional file 1
**Mathematical appendices and data fitting.** (PDF 327 kb)

## Abbreviations

HSC: hematopoietic stem cell
HSPC: hematopoietic stem and progenitor cell
MLE: maximum likelihood estimate
VIS: viral vector integration site
