# Supplementary material for: Mechanisms of blood homeostasis: lineage tracking and a neutral model of cell populations in rhesus macaques
Source: BMC Biol. 2015 Oct 20;13:85. doi: 10.1186/s12915-015-0191-8 (PMC4615871; doi:10.1186/s12915-015-0191-8)
Supplement: Additional file 1 — Mathematical appendices and data fitting. (PDF 327 kb) [file 12915_2015_191_MOESM1_ESM.pdf]

Additional Files: Mathematical Appendices and Data Fitting

Here, we provide additional details of our model, including explicit derivations of the sampled clone size distribution, clone size distributions in the steady-state limit, and the effective parameters that accurately describe our data. We also describe the maximum likelihood estimation used to estimate these parameters.

Derivation of sampled clone size distribution:

We first derive an expression for the expected clone size distribution $\langle m_k(t)\rangle$ in a sample of the differentiated blood, as given by Eq. 6. Define $s_{j\ell}$ to be the number of cells sampled from the $j^{\rm th}$ clone of those that are represented by ℓ cells. At any time, the probability that the configuration $s_{j\ell}$ is observed in a sample of S cells can be written

$$P(\{s\}) = S!\prod_{\ell=1}^{N_{\rm d}}\prod_{j=1}^{y_\ell}\binom{\ell}{s_{j\ell}}\frac{\delta_{X,S}}{N_{\rm d}^{s_{j\ell}}}, \tag{A1}$$

where $X \equiv \sum_{\ell=1}^{N_{\rm d}}\sum_{j=1}^{y_\ell} s_{j\ell}$, the factor $N_{\rm d}^{-s_{j\ell}}S!$ represents the probability that $s_{j\ell}$ cells were drawn from the $N_{\rm d}$ within a sample of S cells, and $\binom{\ell}{s_{j\ell}}$ is the number of ways of drawing $s_{j\ell}$ cells. Finally, the last Kronecker $\delta-$function forces the sum over all $s_{j\ell}$ to equal the total number of cells sampled and sequenced. In any particular sample, the number of clones with size k is exactly

$$m_k = \sum_{n=1}^{N_{\rm d}}\sum_{m=1}^{y_n}\delta_{k,s_{mn}}. \tag{A2}$$

The expected value of this quantity is

$$\langle m_k(t)\rangle = \sum_{\{s\}}P(\{s\})\sum_{\ell=0}^{N_{\rm d}}\sum_{j=1}^{y_\ell}\delta_{k,s_{j\ell}}, \tag{A3}$$

which can be found by using the generating function $G(z,t) \equiv \sum_{k=0}^{\infty}\langle m_k(t)\rangle z^k$:

$$\begin{aligned} G(z,t;S) &= \sum_{\{s\}}P(\{s\})\sum_{\ell=0}^{N_{\rm d}}\sum_{j=1}^{y_\ell} z^{s_{j\ell}} \\ &= \frac{\partial}{\partial\beta}\sum_{\{s\}}P(\{s\})\exp\left[\beta\sum_{\ell=0}^{N_{\rm d}}\sum_{j=1}^{y_\ell}z^{s_{j\ell}}\right]\Bigg|_{\beta=0}. \end{aligned} \tag{A4}$$

After using the Fourier representation of the Kronecker $\delta-$function in Eq. A1, $2\pi\delta_n = \int_0^{2\pi} e^{iqn}{\rm d}q$, we can further reduce the generating function to

$$\begin{aligned} G(z,t;S) &= \frac{\partial}{\partial\beta}\int_0^{2\pi}\frac{{\rm d}q}{2\pi}S!e^{-iqS}\prod_{\ell=0}^{N_{\rm d}}\prod_{j=1}^{y_\ell}\left[\sum_{s=0}^{\ell}\binom{\ell}{s}\frac{e^{iqs}}{N_{\rm d}^s}e^{\beta z^s}\right]_{\beta=0} \\ &= \sum_{\ell=0} y_\ell S!\int_0^{2\pi}\frac{{\rm d}q}{2\pi}e^{-iqS}\frac{\sigma(\ell,z)}{\sigma(\ell,1)}\prod_{i=1}[\sigma(i,1)]^{y_i}, \end{aligned} \tag{A5}$$

where

$$\sigma(\ell,z) \equiv \sum_{s=0}^{\ell}\binom{\ell}{s}\frac{e^{iqs}}{N_{\rm d}^s}z^s. \tag{A6}$$

Note that $\sigma(\ell,z) \equiv (1+ze^{iq}/N_{\rm d})^\ell$, and that $\prod_{i=1}[\sigma(i,1)]^{y_i} \approx (1+e^{iq}/N_{\rm d})^{N_{\rm d}}$. Since $N_{\rm d} \gg S \gg 1$, and $N_{\rm d} \sim 10^9 - 10^{10}$, we can take the large $N_{\rm d}$ limit before the large S limit to find $(1+e^{iq}/N_{\rm d})^{N_{\rm d}} \approx \exp\left[e^{iq}\right]$, $\sigma(\ell,z) \approx \exp[e^{iq}z\ell/N_{\rm d}]$, and

$$G(z,t;S) \approx \sum_{\ell=0}^{\infty} y_\ell S! \int_0^{2\pi} \frac{\mathrm{d}q}{2\pi} \exp\Big[A_\ell(z)e^{iq}\Big]\, e^{-iqS}, \tag{A7}$$

where $A_\ell(z) = 1 + \ell(z-1)/N_\mathrm{d}$. Note that the integral is simply Euler's integral for $1/\Gamma(S+1)$. Namely, we find

$$\begin{aligned}\int_0^{2\pi} \frac{\mathrm{d}q}{2\pi} \exp\Big[A_\ell(z)e^{iq}\Big]\, e^{-iqS} &= \frac{iA_\ell^S(z)}{2\pi} \int_\mathcal{C} \mathrm{d}\xi e^{\xi} \xi^{-(S+1)} \\ &= \frac{A_\ell^S(z)}{\Gamma(S+1)}. \end{aligned} \tag{A8}$$

Since $A_\ell^S = (1 + \ell(z-1)/N_\mathrm{d})^S = (1 + (S/N_\mathrm{d})\ell(z-1)/S)^S \approx e^{\ell S(z-1)/N_\mathrm{d}}$ for $S \gg 1$, we find, for $\varepsilon \equiv S/N_\mathrm{d} \ll 1$,

$$G(z,t;S) = \sum_{\ell=0}^{N_\mathrm{d}} y_\ell(t) A_\ell^S(z) \approx \sum_{\ell=0} y_\ell(t) e^{\ell\varepsilon(z-1)}, \tag{A9}$$

Next, we define the fraction of clones of size $1 \leq q \leq S$ or less. This distribution includes unrepresented or lost clones, and is defined as $F(q,t) \equiv \sum_{i=0}^{q} \langle m_i(t)\rangle$. By using Eq. A9 and the definition of $G(z,t)$, we find

$$F(q,t) \equiv \sum_{j=0}^{q} \sum_{n=0}^{\infty} e^{-n\varepsilon} \frac{(n\varepsilon)^j}{j!} y_n(t). \tag{A10}$$

The expected clone size distribution is thus defined as

$$\langle m_k(t)\rangle = F(k,t) - F(k-1,t) = \sum_{\ell=0}^{\infty} e^{-\ell\varepsilon} \frac{(\ell\varepsilon)^k}{k!} y_\ell(t). \tag{A11}$$

In general, further development of $F(k,t)$ and $\langle m_k(t)\rangle$ requires numerical solution of $c_k(t)$ and $y_\ell(t) = \sum_{k=0}^{\infty} y_\ell^{(k)}(t)$. The time-dependence of $F(q,t)$ is further complicated by the time-dependence of $\varepsilon(t) = S/N_\mathrm{d}(t)$, requiring the solution to Eq. 4.
The variability of m_k due to sampling can be also estimated by calculating $\langle m_k m_{k'}\rangle$, which we write as

$$\langle m_k m_{k'}\rangle = \sum_{\{s\}} P(\{s\}) \sum_{\ell=0}^{N_\mathrm{d}} \sum_{j=1}^{y_\ell} \delta(s_{j\ell} - k) \sum_{\ell'=0}^{N_\mathrm{d}} \sum_{j'=1}^{y_{\ell'}} \delta(s_{j'\ell'} - k'). \tag{A12}$$

This calculation requires evaluation of the two-dimensional generating function

$$G(z,z',t) = \sum_{k,k'} \langle m_k(t) m_{k'}(t)\rangle z^k z'^{k'}. \tag{A13}$$

After using Eq. A1 for $P(\{s\})$ in Eq. A12 and performing some algebra, we find

$$G(z,z',t) = \sum_{\ell,\ell'} A_{\ell,\ell'}^S(z,z') y_\ell(t) y_{\ell'}(t) - \sum_\ell B_\ell^S(z,z') y_\ell(t) + \sum_\ell C_\ell^S(zz') y_\ell(t), \tag{A14}$$

where

$$A_{\ell,\ell'}(z,z') = 1 + \frac{\varepsilon(z'-1)\ell'}{S} + \frac{\varepsilon(z-1)\ell}{S}$$

$$B_{\ell}(z,z') = 1 + \frac{\varepsilon(z'-1)\ell}{S} + \frac{\varepsilon(z-1)\ell}{S}$$

$$C_{\ell}(zz') = 1 + \frac{\varepsilon(zz'-1)\ell}{S}.$$

Using $(1+x/S)^S \approx e^x$, and expanding in powers of z and z', we find

$$\begin{aligned}\langle m_k(t) m_{k'}(t)\rangle = & \left[\sum_{\ell} y_\ell(t) e^{-\varepsilon\ell}\frac{(\varepsilon\ell)^k}{k!}\right]\left[\sum_{\ell'} y_{\ell'}(t) e^{-\varepsilon\ell'}\frac{(\varepsilon\ell')^{k'}}{k'!}\right] \\ & -\sum_{\ell} y_\ell(t) e^{-2\varepsilon\ell}\frac{(\varepsilon\ell)^k}{k!}\frac{(\varepsilon\ell)^{k'}}{k'!} + \sum_{\ell} y_\ell(t) e^{-\varepsilon\ell}\frac{(\varepsilon\ell)^k}{k!}\delta_{k,k'}.\end{aligned} \tag{A15}$$

The diagonal variance is simply

$$\begin{aligned}\langle m_k^2(t)\rangle - \langle m_k(t)\rangle^2 &= \sum_{\ell} y_\ell(t) e^{-\varepsilon\ell}\frac{(\varepsilon\ell)^k}{k!}\left[1 - e^{-\varepsilon\ell}\frac{(\varepsilon\ell)^k}{k!}\right] \\ &= \langle m_k(t)\rangle - \sum_{\ell} y_\ell(t)\frac{e^{-2\varepsilon\ell}(\varepsilon\ell)^{2k}}{(k!)^2}.\end{aligned} \tag{A16}$$

The second term is much smaller than the first except for very small values of k. Therefore, the relative fluctuation in m_k due to sampling is

$$\frac{\sqrt{\langle m_k^2(t)\rangle - \langle m_k(t)\rangle^2}}{\langle m_k(t)\rangle} \lesssim \frac{1}{\sqrt{\langle m_k(t)\rangle}}, \tag{A17}$$

explicitly indicating that the relative fluctuations in the measured number of clones of size k decreases as the square-root of its expected value.

Steady-state solution:
As was discussed, the total peripheral blood population in the animals recovered quickly, usually within a few weeks after transplantation. Moreover, from our data, the overall qualitative shape of the clone size distribution also reaches steady-state only after a few months post-transplant, with no discernible systematic time-dependence. Therefore, we consider the steady-state solutions to our model (Eqs. 1 and 5). Henceforth, all quantities will be assumed to be those at steady-state. First, we can start from $n = 1$ and inductively solve for the steady-state form of Eqs. 5 to find

$$y_n = \sum_{k=0}^{\infty} y_n^{(k)} = \sum_{k=0}^{\infty}\frac{(wk)^n}{n!}e^{-wk}c_k,\ \ w = \frac{(1+\eta)\omega}{\mu_{\mathrm{d}}}. \tag{A18}$$

Before this solution can be effectively used in Eq. A10, an explicit expression for the steady-state progenitor clone size distribution c_k is needed.
The total steady-state progenitor population is given by the solution to $\alpha C + [r(N_{\mathrm{p}}) - \mu]N_{\mathrm{p}} = 0$. The population-limited growth rate is given by Eq. 3 and the steady-state progenitor cell population is explicitly

$$\frac{N_{\mathrm{p}}}{K} = \frac{1}{2}\left[\frac{\alpha}{\mu}\frac{U+C}{K} + \frac{p}{\mu} - 1 + \sqrt{\left(\frac{\alpha}{\mu}\frac{U+C}{K} + \frac{p}{\mu} - 1\right)^2 + \frac{4\alpha}{\mu}\frac{U+C}{K}}\right], \tag{A19}$$

where $\mu = \mu_{\mathrm{p}} + \eta\omega$. After using Eq. A19 in Eq. 3, we find explicitly

$$r(N_{\rm p}) = \frac{2p}{\frac{\alpha}{\mu}\frac{U+C}{K} + \frac{p}{\mu} + 1 + \sqrt{\left(\frac{\alpha}{\mu}\frac{U+C}{K} + \frac{p}{\mu} - 1\right)^2 + \frac{4\alpha}{\mu}\frac{U+C}{K}}} < \mu. \tag{A20}$$

The total differentiated cell population found from $\dot{N}_{\rm d} = (1+\eta)\omega N_{\rm p} - \mu_{\rm d} N_{\rm d} \approx 0$ is $N_{\rm d} = (1+\eta)\omega N_{\rm p}/\mu_{\rm d} \equiv w N_{\rm p}$. Upon using these expressions in the steady-state limit of Eq. 1, we obtain

$$\begin{aligned} c_{k\geq 1} &= \frac{aC}{k!}\frac{\bar{r}^k(1-\bar{r})^a}{(a+k)}\prod_{\ell=1}^{k}[a+\ell], \\ c_0 &= C - \sum_{k=1}^{\infty} c_k = C(1-\bar{r})^a, \end{aligned} \tag{A21}$$

where

$$a \equiv \frac{\alpha}{r} \equiv \frac{\alpha}{\mu\bar{r}} \quad \text{and} \quad \bar{r} \equiv \frac{r(N_{\rm p})}{\mu}. \tag{A22}$$

From these results, the total number of clones in each compartment can be explicitly found:

$$\begin{aligned} C_{\rm p} &= \sum_{k=1}^{\infty} c_k = C\left[1-(1-\bar{r})^a\right], \\ C_{\rm d} &= \sum_{n=1}^{\infty}\sum_{k=1}^{\infty} y_n^{(k)} = \sum_{k=1}^{\infty}(1-e^{-wk})c_k \\ &= C\left[1-\left(\frac{1-\bar{r}}{1-\bar{r}e^{-w}}\right)^a\right]. \end{aligned} \tag{A23}$$

For the total expected number of clones observed in the sample,

$$\begin{aligned} C_{\rm s} &= \sum_{j=1}^{\infty}\langle m_j\rangle = \sum_{j=1}^{\infty}\sum_{\ell=1}^{\infty} e^{-\ell\varepsilon}\frac{(\ell\varepsilon)^j}{j!}y_\ell \\ &= \sum_{k=1}^{\infty}\sum_{\ell=1}^{\infty}(1-e^{-\ell\varepsilon})\frac{(wk)^\ell}{\ell!}e^{-wk}c_k \\ &= \sum_{k=1}^{\infty}(1-e^{-wke^{-\varepsilon}})c_k \\ &= C\left[1-\left(\frac{1-\bar{r}}{1-\bar{r}e^{-w(1-e^{-\varepsilon})}}\right)^a\right], \end{aligned} \tag{A24}$$

where we have explicitly used Eq. A18 for y_ℓ and Eq. A21 for c_k. This result can be further reduced in two limits

$$C_{\rm s} \approx \begin{cases} C\left[1-\left(\frac{1}{\bar{r}w\varepsilon}\right)^a\left(\frac{1-\bar{r}}{2-\bar{r}}\right)^a\right], & \varepsilon w \gg \frac{(1-\bar{r})}{\bar{r}} \\ aC\frac{\varepsilon w\bar{r}}{1-\bar{r}}, & \varepsilon w \ll \frac{(1-\bar{r})}{\bar{r}}. \end{cases} \tag{A25}$$

As expected, the total numbers of clones present in each pool follow the progression

$$C \gtrsim C_{\rm p} \gtrsim C_{\rm d} > C_{\rm s}, \tag{A26}$$

with significant loss of clones due to sampling ($C_{\rm d} \gg C_{\rm s}$) only in the second case of Eq. A25 describing sample sizes $S \ll \mu_{\rm d} N_{\rm d}(1-\bar{r})/(\omega\bar{r})$. Note that for clone sizes $k \gg a$, c_k in Eq. A21 can be approximated by

$$c_{k\gg a} \approx A k^{a-1} \bar{r}^k \left[1 - \frac{a(1-a)}{2k} + O\left(\frac{1}{k^2}\right)\right], \tag{A27}$$

where

$$A \equiv \frac{aC}{\Gamma(a+1)} (1-\bar{r})^a. \tag{A28}$$

Finally, in steady-state, using Eq. A18 in Eq. A10, we find the cumulative clone size distribution

$$\begin{aligned} F(q) &= \sum_{j=0}^{q} \sum_{\ell=0}^{\infty} \sum_{k=0}^{\infty} \frac{(\ell\varepsilon)^j}{j!} \frac{(wk)^\ell}{\ell!} e^{-\ell\varepsilon} e^{-wk} c_k \\ &\equiv \sum_{\ell=0}^{\infty} \sum_{k=0}^{\infty} \frac{\Gamma(q+1, \ell\varepsilon)}{\Gamma(q+1)} \frac{(wk)^\ell}{\ell!} e^{-wk} c_k \\ &\approx \sum_{k=0}^{\infty} \frac{\Gamma(q+1, \varepsilon wk)}{\Gamma(q+1)} c_k, \end{aligned} \tag{A29}$$

where a steepest descents approximation in $\varepsilon \ll 1$ was used to derive the final approximation. The expected cumulative clone size frequency $Q(q)$ is obtained by subtracting off the unrepresented clones $\langle m_0 \rangle \equiv F(0)$ and normalizing by the total expected number of clones $C_{\rm s} = F(S) - F(0)$.

A number of numerical procedures can be used to evaluate $Q(q)$ using the final approximation in Eq. A29. For large values of q, and small εw, the ratio of $\Gamma-$functions is near unity for $k \lesssim (q+1)/(\varepsilon w)$, and quickly decreases to zero for larger k. One approach for numerically evaluating $F(q)$ is to explicitly separate small q and small k terms in the sum. For small k, the exact form of c_k should be used. For larger k, the asymptotic form (Eq. A27) can be used and the sum can be approximated as an integral. We find that even for small values $q \approx 5$, this approximation results in a relative error $< 1\%$. Even the crudest approximation of replacing the sum by the integral

$$F(q) \approx \sum_{k=0}^{\infty} \frac{\Gamma(q+1, \varepsilon wk)}{\Gamma(q+1)} c_k \approx c_0 + \int_1^{(q+1)/(\varepsilon w)} c_k {\rm d}k, \tag{A30}$$

and using the asymptotic form Eq. A27, provides a reasonable estimate of $F(q)$. This rough approximation also provides an informative analytical expression:

$$F(q) \approx c_0 + \frac{A}{\ln^a(1/\bar{r})} \left[\Gamma\left(a, \ln\left(\frac{1}{\bar{r}}\right)\right) - \Gamma\left(a, \frac{(q+1)}{\varepsilon w} \ln\left(\frac{1}{\bar{r}}\right)\right)\right]. \tag{A31}$$

This approximation shows that our distributions depend most strongly on only $a = \alpha/r$ and $R = (\varepsilon w)/\ln(1/\bar{r})$ (Eq. 10). Since $\bar{r} \approx 1 - \alpha(U+C)/(K(p-\mu))$ is only very slightly smaller than unity, $\ln(1/\bar{r})$ is a small positive number and $A/\ln^a(1/\bar{r}) \approx aC$. Since $\langle m_q \rangle = F(q) - F(q-1)$, an approximate form useful for estimating the clone size distribution is

$$\langle m_q \rangle \approx aC\left[\Gamma(a, q/R) - \Gamma(a, (q+1)/R)\right]. \tag{A32}$$

Within physiologically-relevant regimes, our data can be well-fitted to $\langle m_k \rangle$ by varying just a and R. The other physiological parameters, $K, p, \mu, U+C$, etc., are then related to each other through the most likely numerical values a^* and R^* found from fitting the data.

Consider the total number of active HSC cells, $U+C$, and the ratio of the rate of HSC differentiation to the rate of self-renewal of progenitor cells, α/p. Once the best fit parameters a^* and R^* have been estimated from fitting clonal frequency distributions, $U+C$ and α/p can be expressed in terms of K, S, and $\Delta \equiv p/\mu - 1$. Note that S, the number of sequencing reads detected in each sample, is an experimentally determined parameter.

To find these relationships, we first assume that $\varepsilon w/R^* = (S/N_{\rm d})(\omega/\mu_{\rm d})/R^* = S/(R^* N_{\rm p}) \ll 1$ and $S/(R^* K) \ll 1$. By using the definition of R, we find $\bar{r} = e^{-S/(R^* N_{\rm p})} \approx 1 - S/(R^* N_{\rm p})$. Since $\bar{r} = (\Delta+1)K/(N_{\rm p}+K)$ also, these two independent expressions for $\bar{r}$ furnish a quadratic equation for $N_{\rm p}$ in terms of R^*. After comparing the positive root of this equation to the definition of the steady-state progenitor population $N_{\rm p}$ (Eq. A19), we find

$$\left(\frac{\alpha}{p}\right)\left(\frac{U+C}{K}\right) \approx \frac{S}{(\Delta+1)R^* K}. \tag{A33}$$

Eq. A33 can be then used to find an expression for $N_{\rm p}$ that is independent of α/p and $U+C$. Using this form of $N_{\rm p}$ in the definition $a=\alpha/r=(\alpha/p)\left[N_{\rm p}/K+1\right]$, we find an explicit expression for the best-fit value $\alpha/p=a^{*}K/(N_{\rm p}+K)$. Further assuming that $S/(R^{*}K)\ll\Delta$, we find

$$\left(\frac{\alpha}{p}\right)\approx\frac{a^{*}}{\Delta+1}\left[1-\frac{1}{\Delta}\left(\frac{S}{R^{*}K}\right)+\ldots\right]. \tag{A34}$$

Note that to lowest order, α/p can be estimated from a^{*} and $\Delta=p/\mu-1$. Finally, substituting $(\alpha/p)\approx a^{*}/(\Delta+1)$ from solving Eq. A34 into Eq. A33, we find

$$U+C\approx\frac{S}{a^{*}R^{*}}, \tag{A35}$$

which is independent of K and Δ. Note that these parameters can be extracted out of the many parameters in the model because of the limiting values of $\bar{r}\lesssim 1$ and $\varepsilon w\ll 1$. Our model allows one to make predictions on the number of expected clones in each pool, $C_{\rm p}, C_{\rm d}$, and the measured $C_{\rm s}$ (Eqs. A23-A24), as well as expected clone size distributions (Eq. A32) as functions of sampling fraction ε, turnover rate w, effective differentiation rate a, and effective growth rate $\bar{r}$. However, from the functional forms of $C_{\rm p}, C_{\rm d}, C_{\rm s}$, and because $\bar{r}\approx 1$, the numerical determination of the number of clones in each pool is highly sensitive to high values of R and low values of a.

Expected clone size distributions from stochastic clones sizes:
Here, we explicitly show how the neutral assumption (identical transition rates and fitness for all clones) of our populations allows mean-field equations for the expected clone size distribution to be derived from considerations of the stochastic dynamics of an individual clone. Analysis of individual clones is more natural in settings where each clone can be easily isolated and imaged, such as in epidermal systems and geometries [42, 54, 55]. An important feature of our neutral model is that the steady-state clone size distribution depends on only the value of the effective growth rate at steady-state and not on the specific form of the regulation. In other words, the *relative* sizes of neutral clones are independent of the growth law common to all clones. Therefore, we first consider the corresponding birth-death process of a single isolated clone in the presence of constant immigration occurring at rate α. The master equation for the probability $p_k(t)$ of a single clone containing k progenitor cells is

$$\frac{\mathrm{d}p_k(t)}{\mathrm{d}t}=\alpha\left[p_{k-1}-p_k\right]+r\left[(k-1)p_{k-1}-kp_k\right]+\mu\left[(k+1)p_{k+1}-kp_k\right], \tag{A36}$$

where in our application, $\mu\equiv\mu_{\rm p}+\eta\omega$. If the growth rate r is assumed constant and independent of k, an analytic expression expressed in terms of the corresponding generating function $\phi(z,t)\equiv\sum_{k=0}^{\infty}p_k z^k$ [45, 56]:

$$\phi(z,t)=\left[\frac{(1-\bar{r})}{(1-\bar{r}z)-\bar{r}(1-z)e^{-\mu(1-\bar{r})t}}\right]^{a}. \tag{A37}$$

We now identify $c_k(t)$ with C times the probability that any independent clone is of size k. Thus,

$$\sum_{k=0}c_k z^k\simeq C\phi(z,t) \tag{A38}$$

and the variability in clone sizes arises from the variability of the times of differentiation of HSC cells to create progenitor cells of different lineages. In the $t\to\infty$ steady-state limit, we find

$$\phi(z,t\to\infty)=\left(\frac{1-\bar{r}}{1-\bar{r}z}\right)^{a}=(1-\bar{r})^{a}\sum_{k=0}^{\infty}\frac{\Gamma(k+a)}{\Gamma(a)k!}(\bar{r}z)^{k}, \tag{A39}$$

in which $\bar{r}=r/\mu$. Thus, the single stochastic clone construction of the expected clone size distribution yields

$$c_k=C(1-\bar{r})^{a}\frac{\Gamma(k+a)\bar{r}^{k}}{\Gamma(a)k!}\approx C(1-\bar{r})^{a}\frac{\bar{r}^{k}}{k^{1-a}}, \tag{A40}$$

which matches the result in Eq. A27. This derivation explicitly shows that the exponentially distributed initial differentiation times sets the progenitor cell clone size distribution c_k. This distribution is preserved even in the

mean-field setting of the hodograph-transformed model described by Eq. 1 and is independent of the specific form chosen for the growth law r.

HSC self-renewal:
Rather than assuming that HSCs differentiate only asymmetrically, leaving each unique HSC clone unchanged, we now consider the effects of symmetric HSC replication on the measured clone size distribution. We also assume a separate HSC niche with a corresponding carrying capacity $K_{\rm s}$. If we denote x_k as the number of clones in the stem cell niche that is represented by exactly k stem cells,

$$\begin{aligned}\frac{\mathrm{d}x_1}{\mathrm{d}t} &= -(r_{\rm s}+\mu_{\rm s})x_1 + 2\mu_{\rm s}x_2 \\ \frac{\mathrm{d}x_k}{\mathrm{d}t} &= r_{\rm s}\left[(k-1)x_{k-1} - kx_k\right] + \mu_{\rm s}\left[(k+1)x_{k+1} - kx_k\right],\end{aligned} \tag{A41}$$

where the effective growth rate $r_{\rm s}$ is defined by the carrying capacity $K_{\rm s}$, and the total number of stem cells $N_{\rm s}$, labeled and unlabeled, in the stem cell compartment:

$$r_{\rm s}(N_{\rm s}) = p_{\rm s}\left(1 - \frac{N_{\rm s}}{K_{\rm s}}\right). \tag{A42}$$

Here, we have used logistic growth for mathematical convenience and to simply illustrate the insensitivity of the final clone size distribution to the model of HSC differentiation. The total stem cell population is defined as $N_{\rm s}(t) = U_{\rm s}(t) + \sum_{k=1}^{\infty} kx_k(t)$. Upon summing Eqs. A41 and the equation for unlabeled cells, $\dot{U}_{\rm s} = r_{\rm s}U_{\rm s} - \mu_{\rm s}U_{\rm s}$, we find that the total population decouples and obeys

$$\frac{\mathrm{d}N_{\rm s}}{\mathrm{d}t} = r_{\rm s}(N_{\rm s})N_{\rm s} - \mu_{\rm s}N_{\rm s}, \tag{A43}$$

which can be solved exactly, allowing one to find $r_{\rm s}$ explicitly as a function of time. Eqs. A41 can then be solved numerically to find the stem cell clone frequencies in the stem cell compartment. To simplify the calculations and find a tractable solution, we will set $\mu_{\rm s} = 0$ and define a new time variable $\mathrm{d}\tau = r_{\rm s}(t)\mathrm{d}t$. Equations A41 for $x_k(\tau)$ now have constant coefficients and can be solved by using the initial conditions $x_1(\tau = 0) = C_{\rm s}, x_{k>1}(0) = 0$, and the Laplace transforms,

$$s\tilde{x}_1 - C_{\rm s} = -\tilde{x}_1, \quad s\tilde{x}_k = (k-1)\tilde{x}_{k-1} - k\tilde{x}_k. \tag{A44}$$

The solution

$$\tilde{x}(s) = \frac{C_{\rm s}}{s+1}, \quad \tilde{x}_k(s) = \frac{(k-1)C_{\rm s}}{\prod_{j=1}^{k}(s+j)}, \tag{A45}$$

can be inverted to yield

$$x_1(\tau) = C_{\rm s}e^{-\tau}, \quad x_k(\tau) = C_{\rm s}(e^{\tau}-1)^{k-1}e^{-k\tau}. \tag{A46}$$

To transform back to $x_k(t)$, we need to invert

$$\tau(t) = \int_0^t r_{\rm s}(t')\mathrm{d}t' = p_{\rm s}t - \ln(1 + C_{\rm s}(e^{p_{\rm s}t}-1)/K_{\rm s}). \tag{A47}$$

In the steady-state limit, $t \to \infty$ corresponds to $\tau \to \ln(K_{\rm s}/C_{\rm s}) + O(e^{-p_{\rm s}t})$. In this limit, Eq. A46 yields

$$x_k(t\to\infty) = C_{\rm s}f_{\rm s}(1-f_{\rm s})^{k-1}, \quad f_{\rm s} \equiv \frac{C_{\rm s}}{K_{\rm s}}. \tag{A48}$$

The clone numbers in the progenitor cell population are modeled using

$$\begin{aligned}\frac{\mathrm{d}c_1^{(j)}}{\mathrm{d}t} &= \alpha j \frac{x_j}{C_\mathrm{s}}(c_0 - c_1^{(j)}) - (r+\mu_\mathrm{p})c_1^{(j)} + 2\mu_\mathrm{p} c_2^{(j)} \\ \frac{\mathrm{d}c_k^{(j)}}{\mathrm{d}t} &= \underbrace{\alpha j \frac{x_j}{C_\mathrm{s}}(c_{k-1}^{(j)} - c_k^{(j)})}_{\text{HSC asym. differentiation}} + \underbrace{r((k-1)c_{k-1}^{(j)} - kc_k^{(j)})}_{\text{progenitor birth}} + \underbrace{\mu((k+1)c_{k+1}^{(j)} - kc_k^{(j)})}_{\text{progenitor death}},\end{aligned} \tag{A49}$$

where $c_0(t) \equiv C_\mathrm{s} - \sum_{j,\ell=1}^{\infty} c_\ell^{(j)}(t)$ is the total number of clones that do not appear in the progenitor cell pool at time t. Upon summing all the above equations to find the zeroth and first moments of $c_k^{(j)}$, we find

$$\frac{\mathrm{d}}{\mathrm{d}t}\left(\sum_{k=1}^{\infty} c_k^{(j)}(t)\right) = A_j c_0(t) - \mu_\mathrm{p} c_1^{(j)}, \tag{A50}$$

and

$$\frac{\mathrm{d}}{\mathrm{d}t}\left(\sum_{k=1}^{\infty} kc_k^{(j)}(t)\right) = A_j \sum_{k=1}^{\infty} c_k^{(j)} + (r_\mathrm{p}(N_\mathrm{p}) - \mu_\mathrm{p})\sum_{k=1}^{\infty} kc_k^{(j)}(t), \tag{A51}$$

where $A_j(t) = \alpha j x_j(t)/C_\mathrm{s}$. By further adding $\dot{U}_\mathrm{p} = \alpha U_\mathrm{s} + (r_\mathrm{p} - \mu_\mathrm{p})U_\mathrm{p}$ to Eq. A51, we find

$$\frac{\mathrm{d}N_\mathrm{p}}{\mathrm{d}t} = \alpha U_\mathrm{s} + \frac{\alpha}{C_\mathrm{s}} \sum_{j,k=1}^{\infty} jx_j(t)c_k^{(j)}(t) + (r_\mathrm{p}(N_\mathrm{p}) - \mu_\mathrm{p})N_\mathrm{p}(t). \tag{A52}$$

If we assume steady-state in both the stem cell and progenitor cell populations,
$A_j = j\alpha x_j(\infty)/C_\mathrm{s} = j\alpha f_\mathrm{s}(1-f_\mathrm{s})^{j-1}$ and Eq. A50 yields

$$c_1^{(j)} = \frac{A_j}{\mu_\mathrm{p}} c_0 = j\frac{\alpha}{\mu_\mathrm{p}} f_\mathrm{s}(1-f_\mathrm{s})^{j-1} c_0, \tag{A53}$$

which, when used in the steady-state limit of Eq. A51 yields

$$c_k^{(j)} = \frac{c_0 \bar{r}^k}{k!} \prod_{\ell=1}^{k} \left[a_j + (\ell - 1)\right], \tag{A54}$$

where

$$a_j = \frac{jx_j}{C_\mathrm{s}} \frac{\alpha}{r} = jf_\mathrm{s}(1-f_\mathrm{s})^{j-1}\frac{\alpha}{r}. \tag{A55}$$

The coefficient c_0 can now be self-consistently calculated by noting that $c_0 + \sum_{j,k=1}^{\infty} c_k^{(j)} = C_\mathrm{s}$. Upon double-summing Eq. A54, we find $c_0 = C_\mathrm{s}/\mathcal{Z}$, where

$$\mathcal{Z} \equiv 1 + \sum_{j=1}^{\infty}\left[(1-\bar{r})^{-a_j} - 1\right], \tag{A56}$$

and

$$c_k^{(j)} = \frac{C_\mathrm{s}\bar{r}^k}{\mathcal{Z}k!} \prod_{\ell=1}^{k} \left[a_j + (\ell - 1)\right], \tag{A57}$$

The clone numbers in the progenitor pool are thus $c_k = \sum_{j=1}^{\infty} c_k^{(j)}$. Note that when the initial transplantation fills the entire stem cell niche, $f_\mathrm{s} \to 1^-$, $\mathcal{Z} \to (1-\bar{r})^{-a}$, and the only term in Eq. A54 that survives is $j = 1$, leading to our previous result as expected. For the general product in Eq. A54, we can approximate

$$\prod_{\ell=1}^{k} [a_j + (\ell - 1)] \approx (k-1)! \frac{k^{a_j}}{\Gamma(a_j)}, \tag{A58}$$

when $a_j \ll \ln k$. From Eq. A55, we know that a_j is strictly bounded above by α/r and is typically $\lesssim 0.5\alpha/r$ for $f_\mathrm{s} < 0.5$. Since we expect $\alpha/r < 1$, the approximation in Eq. A58 is valid for essentially all values of $k \gtrsim 2$. In order to compute c_k, we perform the sum

$$c_k \approx \frac{C_\mathrm{s}\bar{r}^k}{\mathcal{Z}k} \sum_{j=1}^{\infty} \frac{k^{a_j}}{\Gamma(a_j)}. \tag{A59}$$

By Taylor-expanding in small a_j first, we can further approximate the sum as

$$\sum_{j=1}^{\infty} \frac{k^{a_j}}{\Gamma(a_j)} \approx \frac{a}{f_\mathrm{s}} + \frac{a^2}{f_\mathrm{s}} \frac{(2 - f_\mathrm{s}(2-f_\mathrm{s}))}{(2-f_\mathrm{s})^3} (\gamma + \ln k) + O(a^3) \tag{A60}$$

where $a \equiv \alpha/r$ and $\gamma \approx 0.5772$ is Euler's constant. In order to find an explicit expression for $\mathcal{Z}$, note that $\delta \equiv 1 - \bar{r}$ is typically very small, on the order of $1/K_\mathrm{p}$. Therefore $\mathcal{Z} = 1 + \sum_{j=1}^{\infty} [\delta^{-a_j} - 1]$ can be approximated using $\delta^{-a_j} = \exp[-a_j \ln \delta] \gg 1$ and Laplace's method on the sum. The dominant term in the sum arises for $j^* \approx -1/\ln(1 - f_\mathrm{s})$. Approximating the sum by an integral over a Gaussian centered about j^*, we find

$$\mathcal{Z} \approx \frac{\sqrt{2\pi e r(1-f_\mathrm{s})} \exp\left[\left(\frac{\alpha f_\mathrm{s}}{er}\right) \frac{\ln(1-\bar{r})}{(1-f_\mathrm{s})\ln(1-f_\mathrm{s})}\right]}{\sqrt{\alpha f_\mathrm{s} \ln(1-\bar{r}) \ln(1-f_\mathrm{s})}}. \tag{A61}$$

By using this approximation for $\mathcal{Z}$ and Eq. A60 in Eq. A59, we can find the leading behavior of c_k in the small f_s and $a \ln k \ll 1$ limits,

$$c_k \approx a K_\mathrm{s} (1-\bar{r})^{a/e} \sqrt{\frac{a}{2\pi e} \ln\left(\frac{1}{1-\bar{r}}\right)} \frac{\bar{r}^k}{k}. \tag{A62}$$

This approximation is not good for small a since it suppresses the largeness of the asymptotic parameter $-\ln(1-\bar{r})$. Instead, for small $|a \ln(1-\bar{r}) \ll 1$, we Taylor expand $\exp[-a_j \ln \delta] \approx 1 - a_j \ln \delta$ to find

$$\mathcal{Z} \approx 1 - \frac{a}{f_\mathrm{s}} \ln(1-\bar{r}), \tag{A63}$$

and

$$c_k \approx \left[1 + \frac{a(2 - f_\mathrm{s}(2-f_\mathrm{s}))(\gamma + \ln k)}{(2-f_\mathrm{s})^3}\right] \frac{a K_\mathrm{s}}{1 - a\ln(1-\bar{r})/f_\mathrm{s}} \left(\frac{\bar{r}^k}{k}\right). \tag{A64}$$

Thus, in the limit where the transplanted number of clones $C_\mathrm{s} \ll K_\mathrm{s}$ is much less than the HSC carrying capacity, the marked HSCs greatly expand before reaching K_s; however, the resulting clone size distribution c_k remains qualitatively unchanged.

Maximum Likelihood Estimation:
We start with the counts of unique sequencing reads on the macaque genome. $i.e.$ number of times the read was sequenced. We refer to each unique read as a "clone." Since sequencing of each end of a unique viral sequence is performed independently, we treat the two data sets as independent measurements at each time. The reads are then pooled according to which end of a read was sequenced. For more details of the sequencing and filtering of the reads, see Kim $et\ al.$ [14].

Assume a sequencing run from a particular animal, at a particular time and at one of the sequencing ends, yields n unique clones with $\{q_1, \ldots, q_i, \ldots, q_n\}$ read counts. We calculate the likelihood of observing this data within our model given a particular set of parameter values. Our mathematical model contains three independent parameter combinations:

$$a \equiv \frac{\alpha}{r}, \quad \bar{r} \equiv \frac{r}{\mu}, \quad \text{and} \quad \varepsilon w \equiv \frac{S}{N_{\rm d}} \times \frac{\omega}{\mu_{\rm d}}. \tag{A65}$$

Since the distinguishable clones are otherwise physiologically identical, we associate the distribution of sizes of any particular clone with the expected value of clone size distribution:
$\mathcal{P}(q_i|a, \bar{r}, \varepsilon w) \approx \langle m_{q_i}(a, \bar{r}, \varepsilon w)\rangle = F(q|a, \bar{r}, \varepsilon w) - F(q-1|a, \bar{r}, \varepsilon w)$. The likelihood of the parameters given the detected clone sizes $\{q_1, \ldots, q_n\}$ is then given by:

$$\mathcal{L}(a, \bar{r}, \varepsilon w|\{q_1, \ldots, q_n\}) = \prod_{i=1}^{n} \mathcal{P}(q_i|a, \bar{r}, \varepsilon w). \tag{A66}$$

The most likely parameters are then estimated by numerically maximizing the likelihood over the parameters. However, as shown previously, the distribution of clone sizes depends most strongly on only a and R given by Eqs. 10.
The figures below show normalised and rescaled clone size distributions extracted from granulocyte or peripheral blood mononuclear cell (PBMC) subpopulations of blood from all animals in the original study. The MLE values of a^* and R^* all fall within regimes such that $U + C \sim 10^3 - 10^4$. The fluctuations in $U + C$ are predominantly due to changes in the fraction f at different time points. Such fluctuations are the result of internal dynamics not considered in our model and do not exhibit any discernible trend.

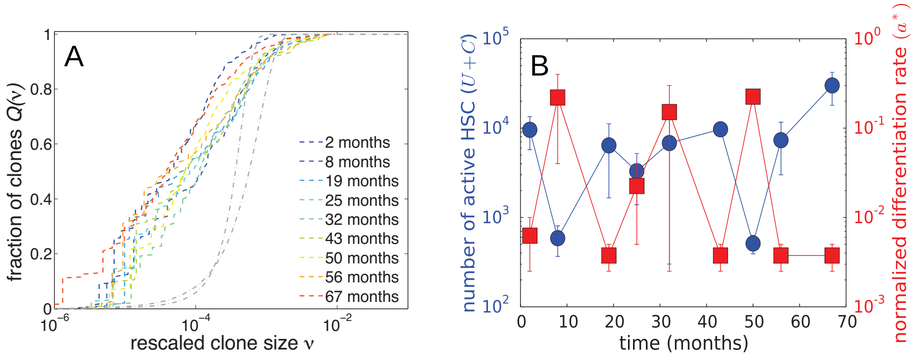

Fig. A1: (A) Rescaled clone size distributions from granulocytes in animal RQ5427. The stationarity in this subpopulation is evident. (B) The MLE fits for $U + C$ (blue circles) and a^* (red squares).

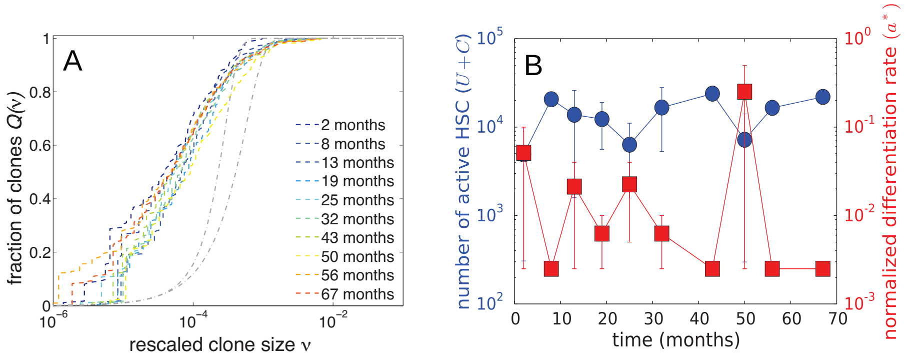

Fig. A2: (A) Rescaled PBMC clone size distributions from animal RQ5427. (B) MLE fits for $U + C$ and a^*.

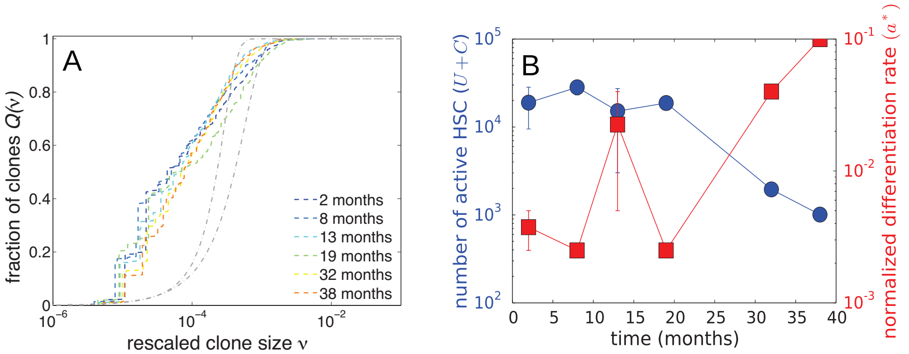

Fig. A3: (A) Rescaled granulocyte clone size distributions from animal RQ3570. (B) MLE fits for $U + C$ and a^*.

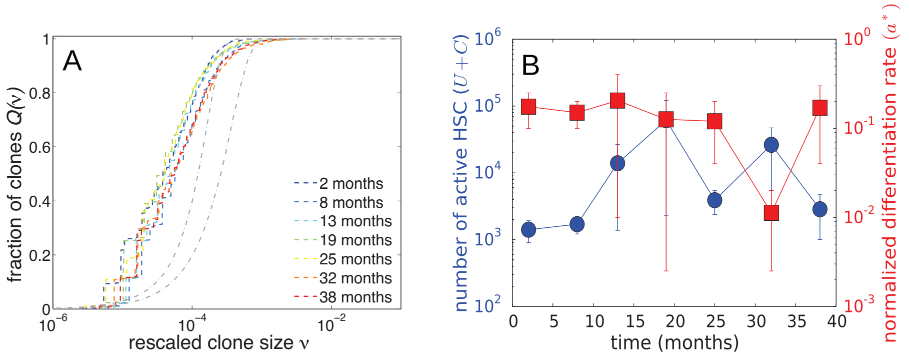

Fig. A4: (A) Rescaled PBMC clone size distributions from animal RQ3570. (B) MLE fits for $U + C$ and a^*.

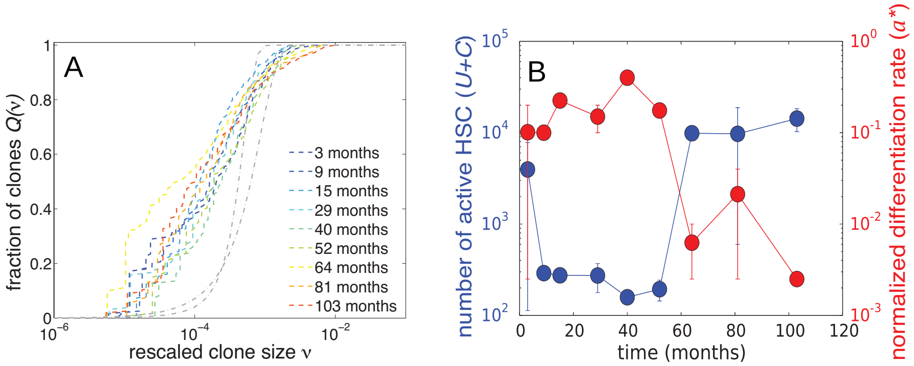

Fig. A5: (A) Rescaled clone size distributions from granulocytes in animal 2RC003. The stationarity in this subpopulation is evident. (B) The MLE fits for $U + C$ (blue circles) and a^* (red squares).

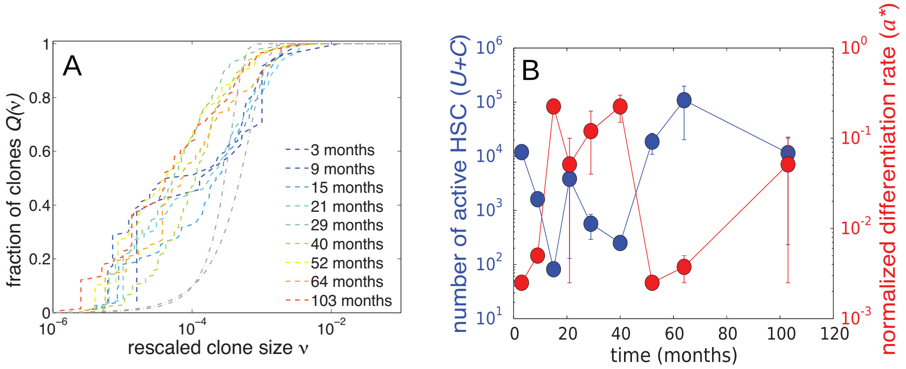

Fig. A6: (A) Rescaled PBMC clone size distributions from animal 2RC003. (B) MLE fits for $U + C$ and a^*.

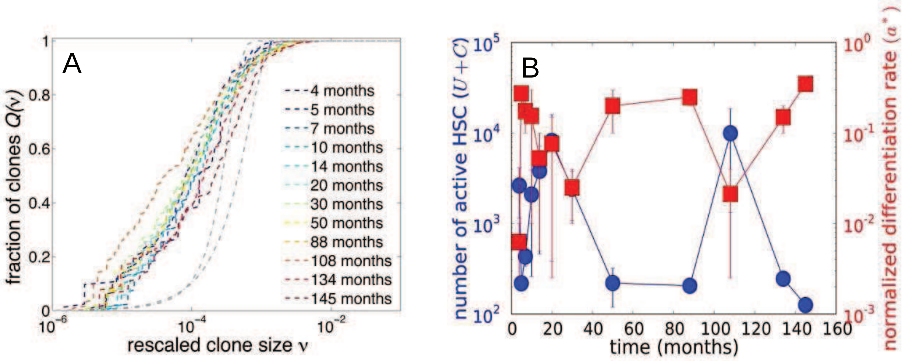

Fig. A7: For animal 95E132 clone sizes were measured only for within the total peripheral blood cells (PBC) population. (A) Rescaled clone size distributions from animal 95E132. (B) MLE fits for $U + C$ and a^* from the combined PBC clone size distributions.

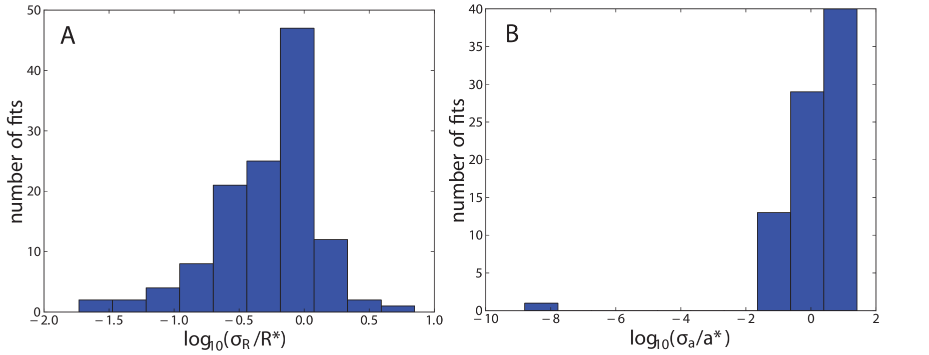

Fig. A8: (A) Distribution of $\log(\sigma_R/R^*)$ values across all data sets. (B) Distribution of $\log(\sigma_a/a^*)$ values.

For completeness, we also calculate a rough goodness-of-fit metric. We do this by calculating the "diagonal" curvatures of the likelihood function $\partial^2\mathcal{L}/\partial R^2$ and $\partial^2\mathcal{L}/\partial a^2$ evaluated at the maximum (R^*, a^*). Upon defining

$$\sigma_R = \left[\left(\frac{\partial^2\mathcal{L}}{\partial R^2}\right)_{R^*,a^*}\right]^{-1/2}, \quad \sigma_a = \left[\left(\frac{\partial^2\mathcal{L}}{\partial a^2}\right)_{R^*,a^*}\right]^{-1/2}, \tag{A67}$$

a goodness-of-fit can be measured through the distribution of the values of the Fano factors σ_a/a^* and σ_R/R^* obtained by fitting each clone size distribution at each time point. The distributions of the logarithm of σ_a/a^* and σ_R/R^* (sampled from fitting at all times points for all animals) are plotted below. We see that the fitting for R at most time points is reasonably good, but that some of the fits, particularly for the small values of a^*, are not particularly well-conditioned.

Additional file references

54. Klein AM, Doup DP, Jones PH, Simons BD. Mechanism of murine epidermal maintenance: cell division and the voter model. Phys Rev E. 2008;77:031907.

55. Klein AM, Nikolaidou-Neokosmidou V, Doup DP, Jones PH, Simons BD. Patterning as a signature of human epidermal stem cell regulation. J R Soc Interface. 2011;8:181524.

56. Chou T, Wang Y. Fixation times in differentiation and evolution in the presence of bottlenecks, deserts, and oases. J Theor Biol. 2015;372:6573.
